# An *in silico* analysis of acquired antimicrobial resistance genes in *Aeromonas* plasmids

**DOI:** 10.3934/microbiol.2020005

**Published:** 2020-03-16

**Authors:** Ogueri Nwaiwu, Chiugo Claret Aduba

**Affiliations:** 1 School of Biosciences, University of Nottingham, Sutton Bonington Campus, United Kingdom; 2 Department of Science Laboratory Technology, University of Nigeria, Nsukka, Nigeria

**Keywords:** Aeromonas, acquired antimicrobial resistance, plasmids, GC content, multiple antimicrobial resistance, predicted multidrug resistance, Colistin

## Abstract

Sequences of 105 *Aeromonas* species plasmids were probed for acquired anti-microbial resistance (AMR) genes using a bioinformatics approach. The plasmids showed no positive linear correlation between size and GC content and up to 55 acquired AMR genes were found in 39 (37%) plasmids after *in silico* screening for resistance against 15 antibiotic drug classes. Overall, potential multiple antibiotic resistance (p-MAR) index ranged from 0.07 to 0.53. Up to 18 plasmids were predicted to mediate multiple drug resistance (MDR). Plasmids pS121-1a (*A. salmonicida*), pWCX23_1 (*A. hydrophila*) and pASP-a58 (*A. veronii*) harboured 18, 15 and 14 AMR genes respectively. The five most occurring drug classes for which AMR genes were detected were aminoglycosides (27%), followed by beta-lactams (17%), sulphonamides (13%), fluoroquinolones (13%), and phenicols (10%). The most prevalent genes were a sulphonamide resistant gene *Sul1*, the gene *aac (6′)-Ib-cr* (aminoglycoside 6′-N-acetyl transferase type Ib-cr) resistant to aminoglycosides and the *blaKPC-2* gene, which encodes carbapenemase-production. Plasmid acquisition of AMR genes was mainly inter-genus rather than intra-genus. Eighteen plasmids showed template or host genes acquired from *Pseudomonas monteilii*, *Salmonella enterica* or *Escherichia coli*. The most occurring antimicrobial resistance determinants (ARDs) were beta-lactamase, followed by aminoglycosides acetyl-transferases, and then efflux pumps. Screening of new isolates *in vitro* and *in vivo* is required to ascertain the level of phenotypic expression of colistin and other acquired AMR genes detected.

## 1. Introduction

The genus *Aeromonas* are ubiquitous and colonize aquatic and terrestrial environments. They can be found in different sources, like soils, freshwater, plants, fruits, vegetables, birds, fish, reptiles, and amphibians [1],[2]. Species of *Aeromonas* are Gram-negative and can cause food spoilage or infection in animals and humans [3]. They are also referred to as emerging foodborne pathogens for over four decades, which indicates that the pathogenic potential of the genus has not been fully established. Its occurrence in urban-associated environmental waters [4]–[6] is worrying and could be a source of gastroenteritis. Species of *Aeromonas* have been implicated in diarrheal illnesses in children, which is aided by exposure to recreational water activities [7]. The contribution of *Aeromonas* spp. to bacterial gastroenteritis has not been comprehensively studied and it has been suggested that further studies of pathogenicity are required [8] because most *Aeromonas* species have some similarities with ubiquitous pathogenic foodborne microorganisms.

The species that have been obtained from human clinical samples were summarized in a review [9], and it includes *A. hydrophila, A. caviae, A. veronii, and A. jandaei*. It also includes *A. schubertii, A. trota*, and *A. eucrenophila*. Other clinical isolates mentioned are *A. enteropelogenes, A. diversa, A. sanarellii, and A. taiwanensis*. The most common clinical presentations of *Aeromonas* are gastrointestinal and wound infections [10],[11]. Another report [12] highlighted that *Aeromonas* isolates can be diverse with a high level of genetic heterogeneity. They also share orthologous genes like the glycerophospholipid-cholesterol acyltransferase gene (*gcat*) used widely in the identification of the genus because it is present in all *Aeromonas* species [13],[14]. Hoel *et al.* [15] have elucidated the characteristics of the genus and current state of research in a comprehensive review. In the review, the debate as to whether *Aeromonas* is a true foodborne pathogen and the multifactorial virulence of the species were highlighted. It was noted that various *Aeromonas* species are commonly recognized as spoilage organisms in seafood and are prevalent in ready to eat seafood.

Presently, the global concern is that anti-microbial drugs are increasingly becoming ineffective due to improper use, which results in secondary or acquired resistance by bacteria that cause infection [16]. To reduce this problem, the World Health Organization (WHO) has championed the global action plan on antimicrobial resistance [17], which aims at reducing antibiotic resistance worldwide. The spread of resistance is aided by human and animals activities and a consensus is that dissemination of antimicrobial resistance is mediated by mobile genetic elements like plasmids. According to a previous report [18], the growing trend of plasmid-mediated resistance to antimicrobial classes of critical importance is due to the emergence of epidemic plasmids, which can rapidly disseminate resistance genes in humans and animals.

Plasmid-borne antimicrobial resistance has been found in *Aeromonas* spp. from isolated freshwater animals [19]. Resistance to water treatment chemicals [20] and different antimicrobial drugs have also been found in *A. hydrophila* isolated from different food samples [21] and in marketed marine fish [22]. It is common knowledge that efflux pumps [23]–[25], integrons [26],[17], lateral or horizontal gene transfer [28]–[30] are factors that facilitate AMR spread in prokaryotes.

Many reports [31]–[33] on *Aeromonas* resistance focused on the *in vitro* phenotypic output without any information on the resistant genes present in the organism. It is, therefore, important for periodic review of emerging trends to help generate useful knowledge on the genetic basis of AMR genes in *Aeromonas*, which may be used for better understanding of the aforementioned factors. The full range of antimicrobial resistance genes that have been acquired in plasmids and genomes of *Aeromonas* has not been extensively reported. Thus, more information on acquired AMR genes is important for monitoring of disease epidemiology. This study aimed to use *in silico* methods to ascertain the prevalence of acquired antimicrobial genes in sequences of *Aeromonas* plasmids.

## 2. Materials and methods

### 2.1. Plasmids selection

A total of 105 plasmid sequences found after a search in the collection of NCBI [34], using the search term ‘*Aeromonas*’ in the search browser were used. It included plasmids from *A. salmonicida, A. hydrophila, A. veronii, A.bestiarum, A. caviae, A. sobria* and other unspeciated *Aeromonas* (Table S1). Sequence information showed that they were released between the year 2001 and 2019. This list will continue to grow as more sequences are added to the database. Strains harbouring the plasmids were from diverse sources like sick fish, river sediment, sewage, hospital effluent, human blood and faeces, fish processing facility, and waste treatment plant. The plasmids ranged between 0.002–0.241 Mb in size and had a GC content of 44.7–64%. Size and GC content of plasmid and genomes were recorded from sequence submission information. Information present in the sequence submissions (source, project) can be accessed using accession numbers of all plasmids used which is shown in Table S1. All *Aeromonas* plasmids sequences in the NCBI database at the time the study was carried out were included.

### 2.2. Screening for acquired AMR genes in plasmids

The 105 plasmid sequences extracted from GenBank (Table S1) were used to perform an *in silico* analysis for the presence of acquired antimicrobial resistance genes in ResFinder 2.1 database/webserver [35],[36]. All fifteen antibiotic drug classes in the database were used for screening regardless of the target site. They included aminoglycoside (AG), beta-lactam, colistin, a fluoroquinolone (FQ), fosfomycin, fusidic acid, and glycopeptide. Others included macrolide-lincosamide-streptogramin B (MLS), nitroimidazole, oxazolidinone, phenicol, rifampicin (RP), sulphonamide (SM), tetracycline (TC), and trimethoprim (TP). Although *Aeromonas* is Gram-negative, the inclusion of glycopeptides drug class normally associated with Gram-positive organisms [37] for screening served as control. The sequences were uploaded into the database program and the screening parameters for acquired resistance genes were set to identify resistance genes to all possible 15-drug classes covered in the server. The percent identity was set at 90% as a minimum and perfect alignment was left at 100%. Percent identity was based on the number of nucleotides that are identical between the best matching resistance gene in the database and the corresponding sequence in the plasmid. The minimum length or the number of nucleotides a sequence must overlap a resistant gene to count as a hit was set at the default of 60%. The plasmid with the highest number of AMR genes was tested at the minimum identity setting of 30 and maximum of 100% in the database.

The accession number of the resistance gene in the database, starting contig position of the gene, and predicted phenotype based on the resistance gene were recorded. Another important feature recorded was the alignment high-scoring segment pair (HSP) query length, which is the length of the alignment between the best matching resistance gene and the corresponding sequence in the genome. Good alignment should cover the entire length of the resistance gene in the database.

Potential multiple antibiotic resistance (p-MAR) index was calculated for all the plasmids tested based on the results gained after screening with the 15 drug classes. The index was calculated as reported by others [38],[39], by calculating the ratio of the number of antibiotic classes to which the isolate displayed resistance to the number of antibiotics to which the isolate had been evaluated for susceptibility. Here, the 15 drug classes in the database represented the number of antibiotics classes evaluated. Following the p-MAR analysis, the plasmids were predicted to be multidrug-resistant (MDR), extensively drug-resistant (XDR) or pan drug-resistant (PDR) using the standards developed by Magiorakus et al. [40].

### 2.3. Verification of acquired AMR genes in Aeromonas plasmids

Plasmids with AMR genes were subjected to another analysis on KmerResistance 2.2 database [41],[42]. The scoring method used was by species determination on maximum query coverage, and the host database was set to bacteria organism or plasmid as required. The gene database was set to resistant genes and the identity threshold was left at the default of 70% with a threshold for depth correction set at 10%. The AMR genes shown in the resulting output was recorded and compared with the output of ResFinder database. The template sequence and host organism were also noted.

### 2.4. Probing of plasmids for antibiotic resistance determinants (ARD)

The plasmids in which AMR genes were detected were screened with ResFinderFG 1.0 [43], which is a functional genomics database that identifies a resistance phenotype based on functional metagenomic antibiotic resistance determinants. Settings used were 98% for per cent identity and 60% for minimum query length. Read type chosen was ‘assembled contigs/genomes’ and sequences used were screened for up to 13 ARD families listed in the database.

### 2.5. Statistical Analysis

Principal component analysis (PCA) was performed with XLSTAT [44] using default settings and normalization to show relationships between plasmid size and GC% content and the summary statistics which included mean, standard deviation, correlation (Pearson) were recorded. An overview of historical connections of the plasmids that harboured the most AMR genes was carried out by comparing the similarity between the plasmid sequences of interest and other sequences available in the nucleotide collection of NCBI using the BLAST tool [45]. To ascertain similarities between query sequences and their closest match, the mean pairwise distance was estimated with MEGA X [46] using the maximum composite likelihood model [47] with default settings. Standard error estimates were obtained by toggling to the bootstrap procedure (1000 replicates). All positions containing gaps and missing data were eliminated (complete deletion option) and the number of positions involved in the analysis was noted.

## 3. Results and discussion

### 3.1. Size and GC content of Aeromonas plasmids

**Figure 1.**
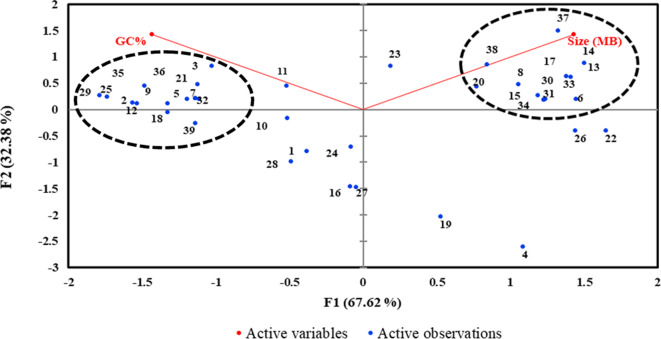
A PCA biplot of variables Size (MB) and GC (%) content for 39 plasmids (1–39 in blue dots). The numbers represent the serial numbers of plasmids listed in Table S2. Dotted black circles show two clusters. Factor one (F1) and factor two (F2) of the PCA show their per cent contribution in brackets.

To determine if there is any association between plasmid size and GC content, a principal component analysis was carried out on the 39 plasmids that harboured AMR genes. The statistics summary (Table S2) showed that plasmids had a minimum of 0.005 and maximum of 0.240 in size (MB) and average was 0.09 MB (± 0.08) whereas the minimum GC content was 46% and maximum 60% with an average of 55 % ± 3.41. Two clusters (dotted lines) appeared to be closely related by size or GC content (Figure 1). However, the negative Pearson's correlation (r = −0.35, α = 0.95) indicate that there is no linear correlation between GC content and plasmid size for the set of plasmids analysed. This may be due to natural variations, which play a key role in base composition and size of bacteria [48].

More studies on GC content and the discovery of new plasmids are required because they are agents of horizontal gene transfer and provide more insights into the natural evolution and propagation of AMR genes [49]. The GC content provides important evolutionary information and can affect genome size in bacteria [50]–[52]. The GC range observed in this study is within the reported average genomic GC- content range of 13–75% among species [53] but higher than the average genome GC content of 50.76% reported for bacteria [54]. It has been suggested that plasmids with higher GC content may be introduced into strains first before plasmids with lower GC content [55] and this may be driven by phylogenetic composition and environmental differences.

### 3.2. AMR genes for different drug classes

**Figure 2.**
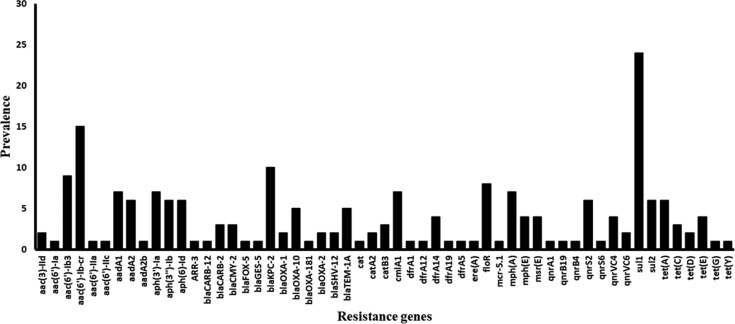
Prevalence of 55 AMR genes found in 39 *Aeromonas* plasmids after in *silico* analysis.

To establish the prevalence of AMR genes, plasmids sequences were screened for AMR gene homologues in ResFinder database, which is a web server that provides a convenient way of identifying acquired antimicrobial resistance genes in completely sequenced isolates [35]. The database displays a limitation, which states that it focuses on acquired genes and does not find chromosomal mutations. It is continuously updated as new resistance genes are identified hence phenotypic confirmation of the presence or absence of detected AMR genes may be required. An investigation [36] found high concordance (99.74%) between phenotypic and predicted whole-genome sequence antimicrobial susceptibility and it was concluded that genotyping using aligned whole-genome sequences is a realistic alternative to surveillance based on phenotypic antimicrobial susceptibility. High alignment accuracy of these sequences is essential for inferring information from multiple alignments [56],[57]. In order to establish if the 90% identity cut off used was the optimum setting, the plasmid (CP022170.1) with the highest AMR genes was tested at 30 and 100% percent identity. It was found that at 30%, 16 AMR genes were detected whereas at 100% 10 were detected which indicates that the default setting of 90% gives an optimal output. Out of the 105 plasmids sequences screened in this study, 39 showed the presence of AMR genes to drug classes tested and no resistance gene was found for five of them namely fosfomycin, fusidic acid, glycopeptide, nitroimidazole and, oxazolidinone. For the other 10 drug classes, several AMR gene homologues were detected. When expressed as a percentage, the five most occurring drug classes in which AMR genes were detected (Table 1) were aminoglycosides (55 out of 208; 27%) followed by beta-lactams (17%), sulphonamides (15%), fluoroquinolones (13%), and phenicols (10%). Fifty-five different acquired AMR genes resistant to the 10 drug classes were detected and specific resistant genes to beta-lactams and aminoglycosides showed the highest prevalence (Table 1). There were AMR genes for drug classes fluoroquinolone phenicol, tetracycline and trimethoprim. Different genes were found for MLS drug class, sulphonamides, colistin and rifampicin. The most prevalent gene was a sulphonamide resistance gene *Sul1*, which was detected 24 times out of 208 (11.5%) observations (Figure. 2). This was followed by the gene *aac (6′)-Ib-cr* (aminoglycoside 6′-N-acetyl transferase type *Ib-cr*) resistant to aminoglycosides and a beta-lactam resistance gene. *blaKPC-2.* This is not a definitive trend due to possible duplications or occurrence of the same gene. However, The *Sul1* resistant gene is common in Gram-negative clinical isolates [58] and is associated with *Pseudomonas* spp. [59]. Both *Sul 1* and *Sul 2* and class 1 integrase gene (*intI1*) has been found in the sediments of urban wetland and it was suggested that they could be used as indicators of environmental contamination with AMR genes [60]. The gene *aac (6′)-Ib-cr* has been found in enterobacterial isolates [61] and is associated with plasmid-mediated quinolone resistance [62]. The *blaKPC-2* gene, which encodes carbapenemase-production, has continued to spread among gram negatives and is now deemed difficult to control [63]. The carbapenemases are also associated with clinically relevant genotypes found in a waste treatment plant and rivers [64].

Antimicrobial genes have been detected in *Aeromonas* spp. by other investigators but fewer genes were usually reported in many reports [65]–[67] when compared to this study. In another report [68], some resistance genes that were detected in this study like *qnrS2* were found whereas other genes like the *bla(CTX-M-3)* were not detected. The high prevalence of *Sul1* and *aac(6′)-Ib-cr* seen in this study is consistent with the highest absolute abundance observed for the two genes in a study of antibiotic gene profiles of a river, which detected *Aeromonas* [69]. The variation may be due in part to *Aeromonas* plasmids diversity or variation with the geographical origin [70]. Plasmids can be an important reservoir of antibiotic resistance genes, which could be exchanged with other bacteria, including human and animal pathogens [71]. The genetic basis of AMR and mechanisms of resistance mediated by plasmids have been reported. Environmental conditions [72] and possession of features that facilitate the global spread of resistance [73] affect plasmid genetic stability. Another suggestion is that spread of plasmids, which possess AMR genes can either be transferred between different genera or derived from a common origin [74]. In this study, the plasmids were detected from strains isolated from various sources, which demonstrates the ubiquity of *Aeromonas* AMR genes in the environment. The sources (Table S1) includes fish, humans, river sediments, snakes and fish processing facility. Others are hospital plumbing and wastewater treatment plants.

**Table 1.** Resistance to different classes of antimicrobial drugs and p-MAR index found in a set of plasmids released between 2001 and 2019. The 39 plasmids listed showed the presence of different number of AMR genes to 10 drug classes . (Aminoglycoside = AG; Beta-lactam = BL; Colistin = CL; fluoroquinolone = FQ;, macrolide-lincosamide-streptogramin B = MLS; Phenicol = PH; Rifampicin = RP; Sulphonamide = SM; Tetracycline = TC; Trimethoprim = TP)

S/n	Organism/Plasmid	AG	BL	CL	FQ	MLS	PH	RP	SM	TC	TP	Total	p-MAR
1	*A. bestiarum* plasmid pAb5S9	2					1		1	1		5	0.27
2	*A. salmonicida* plasmid pRAS3.2									1		1	0.07
3	*Aeromonas* sp. ASNIH1 plasmid pKPC-038c	1	3		1	2			1			8	0.27
4	*Aeromonas* sp. ASNIH2 plasmid pAER-e58e				1							1	0.07
5	*Aeromonas* sp. ASNIH3 plasmid pKPC-cd17		2									2	0.07
6	*Aeromonas* sp. ASNIH3 plasmid pKPC-8e09	1	3		1	2	1		2			10	0.40
7	*Aeromonas* sp. ASNIH4 plasmid pKPC-ac48		2									2	0.07
8	*Aeromonas* sp. ASNIH4 plasmid pAER-f909	1					1		1			3	0.20
9	*Aeromonas* sp. ASNIH5 plasmid pKPC-b21f	1	1		1							3	0.13
10	*Aeromonas* sp. ASNIH7 plasmid pKPC-1ac6	1	2									3	0.13
11	*A. caviae* plasmid pFBAOT6,	1							1	1		3	0.20
12	*A. salmonicida* subsp. *salmonicida* plasmid pRAS3.1									1		1	0.07
13	*A. salmonicida* strain S121 plasmid pS121-1a	4	2		4	2	3		1	1	1	18	0.53
14	*A. salmonicida* strain S44 plasmid pS44-1,	5	1		2	1	2	1	2		1	15	0.53
15	*A. salmonicida* subsp. *salmonicida* A449 plasmid 4,	1					1		1	1		4	0.27
16	*A. sobria* plasmid pAQ2-1				1							1	0.07
17	*A. veronii* strain AVNIH1 plasmid pASP-a58	3	1		2	2	1		3	1	1	14	0.53
18	*A. caviae* strain VBF856 plasmid pIncQ2				1	1						2	0.13
19	*A. caviae* strain VBF856 plasmid pKP3_A		1									1	0.07
20	*A. caviae* GSH8M-1 plasmid pGSH8M-1-1 DNA	2							3			5	0.13
21	*A. caviae* GSH8M-1 plasmid pGSH8M-1-2 DNA	2				1						3	0.13
22	*A. hydrophila* strain D4 plasmid pAhD4-1	1										1	0.07
23	*A. hydrophila* strain AHNIH1 plasmid pASP-135	1	3		1	2	3		3		1	14	0.47
24	*A. hydrophila* plasmid pRA3				2				1	1		4	0.20
25	*A. hydrophila* strain AO1 plasmid pBRST7.6									1		1	0.07
26	*A. hydrophila* plasmid pRA1	1			1							2	0.13
27	*A. hydrophila* plasmid pAQ2-2		1									1	0.07
28	*A. hydrophila* plasmid pAHH01						1					1	0.07
29	*A. hydrophila* plasmid pAHH04								1			1	0.07
30	*A. hydrophila* plasmid pR148	4							1	1		6	0.33
31	*A. hydrophila* strain WCX23 plasmid pWCX23_1	3	4		2	1	2		1	1	1	15	0.53
32	*A. hydrophila* GSH8-2 plasmid pGSH8-2 DNA	2										2	0.07
33	*A. hydrophila* strain WCX23 plasmid unnamed	5	2		2	1	2		1	1	1	15	0.53
34	A. hydrophila strain 23-C-23 plasmid unnamed	1	5		2	1	2		2	1	1	15	0.53
35	Aeromonas hydrophila subsp. hydrophila strain WCHAH045096 plasmid pGES5_045096, complete sequence	2										2	0.13
36	Aeromonas hydrophila subsp. hydrophila strain WCHAH045096 plasmid pKPC2_045096, complete sequence	2										2	0.07
37	Aeromonas hydrophila subsp. hydrophila strain WCHAH045096 plasmid pMCR5_045096, complete sequence	7	1	1	2				1	1	1	14	0.33
38	Aeromonas salmonicida subsp. Salmonicida; pAsa5-3432:	1	1				1		1	2		6	0.33
39	Aeromonas salmonicida subsp. Salmonicida:pRAS3-3432:									1		1	0.07
	Total (%)	55 (27)	34 (17)	1 (0.50)	26 (13)	17 (8.5)	20 (10)	2 (1)	30 (15)	14 (7)	15 (7.5)	208	

#### 3.2.1. Predicted multidrug resistance (p-MAR) index in plasmids of Aeromonas

There are various definitions for the term MDR [75] and that is why we used the interim standard methods [40] for acquired resistance description. Since this study was *in silico*, we limited ourselves to a prediction of MDR, XDR and PDR using the proposed standards. The MAR index was reported to be potential MAR index since an actual *in vitro* study was not carried out. For plasmids, the potential to confer resistance or predicted ability to harbour AMR agents was evaluated. Predicted MDR was seen in 16 plasmids, which harboured resistance genes for three or more drug classes out of 15 tested (Table 1). No XDR or PDR was predicted. The same 16 plasmids had a p-MAR index of 0.20 or more (Table 1) which corresponds to resistance to at least three drug classes. The potential MAR index ranged between 0.07 (resistance to one drug class) and 0.53 (resistance to eight drug classes). Six plasmids had resistance genes to eight drug classes, which included three plasmids of *A. hydrophila*, two of *A. salmonicida*, and a plasmid of *A. veronii* (Table 1). The top three were plasmid pS121-1a (*A. salmonicida*) in which 18 AMR genes were found. Fifteen was detected for plasmid pWCX23_1 (*A. Hydrophila*) and fourteen for plasmid pASP-a58 (*A. veronii*).

Amongst *Aeromonas* spp., multidrug resistance has been reported severally [76],[77] but rarely on the predicted scale found in this study. These findings could assist other researchers to initiate monitoring programs that will help to track the development of XDR or PDR in the environment.

#### 3.2.2 Verification of AMR genes in plasmids and genomes of Aeromonas

Due to variations and mislabeling that could sometimes occur in public databases, it was necessary to confirm the presence of acquired AMR genes found in the set of plasmids of *Aeromonas in* ResFinder database. To this end, the sequences of the 39 plasmids were uploaded into the kmerResistance database for another *in silico* probing for AMR genes. This database overcomes poor quality assembly by using k-mers (fragments of a DNA sequence of length k) to map the raw whole-genome sequence data against reference databases and species [35]. It can also detect host or template genes. Although it is described to be more precise than ResFinder database, the kmerResistance database shows the resistance genes but not the drug classes as an analysis output. Hence, comparisons were limited to resistance genes found in both databases and not the measurement of the sensitivity of both databases *per se*.

The template gene of 18 AMR genes was linked to other Gram-negative organisms (Table S3a) indicating that they could be the source of acquisition. Nine strains were linked to *Pseudomonas monteilii*, eight to *Salmonella enterica* and one to *Escherichia coli*. Also, all the 55 acquired AMR genes found in plasmids within the ResFinder database were detected in kmerResistance database together with a few more possible variants (Table S3b). One colistin resistance gene (*mcr-5*) was found in the ResFinder database whereas two (*mcr-5* and *mcr-3*) were found in kmerResistance database.

Presence of colistin gene homologues in plasmids and genomes is worrying because colistin is an antibiotic of last resort for carbapenem-resistant Gram-negative bacterial infections [78], and there are concerns that colistin resistance may lead to practical pan-antibiotic resistance [79]. A large study [80] with different bacteria found MDR and XDR but no PDR and all gram-negative bacteria were sensitive to colistin. In this study, the observation of colistin genes in both databases is consistent with increasing detection of strains carrying the gene in *Aeromonas* and other bacteria [81],[82]. The slight variation observed with the databases used confirms that the program used for *in silico* probing affects the output. Bioinformaticians have developed many tools to help interpret a large pool of molecular data [83],[84] but confirmation in the laboratory is still very essential for any *in silico* findings.

### 3.3. Antibiotic resistance determinants in Aeromonas plasmids

To determine ARDs, all plasmids which showed the presence of AMR genes were screened. Eight ARD families were detected. The most occurring was beta-lactamase, followed by aminoglycosides acetyl-transferases, and then efflux pumps (Table S4). Others included tet efflux, chloramphenicol acetyl-transferases, aminoglycosides nucleotidyl-transferases, dihydrofolate reductase and the quinolone resistance family. The analysis which found beta-lactamase as the most occurring is consistent with a previous report [2] which highlighted that the inherent presence of beta-lactamase in species of Aeromonas is problematic. Bacterial multidrug efflux pumps are an important class of resistance determinant and if controlled, it could be used to reduce the spread of AMR in the environment. A previous report showed that A. hydrophila can attach to water tanks [85] and this may be aided by genetic determinants mainly located on mobilizable plasmids of Aeromonas spp in the aquatic environment [86]. The Aeromonas strains that harbour up to 18 AMR genes detected in this study may be good candidates for further detailed investigations on ARDs.

### 3.4. Statistical analysis of sequences

To ascertain a holistic prevalence of the plasmids with AMR genes, the sequences of plasmids that harboured the highest number of AMR genes in different *Aeromonas* spp. (Table 1) and the beta-lactam *blaCEPH-A3* (AY112998) resistant gene, which has been found in several studies [87], [88] were used to perform a BLAST® search on the NCBI database. The first 100 hits [See supplementary files] for the *blaCEPH-A3* gene emphasized the orthologous nature of the gene because homologues were found in up to eight different *Aeromonas* species. Only sequences found in *A. veronii* showed a 100 % for query coverage and identity match to the query sequence. Divergence of orthologous genes may explain the evolution of bacterial populations [89] and it is important in the assessment of transfer methods for comparative genomics [90]. Therefore, more studies on this gene may help the understanding of *Aeromonas* AMR genes divergence.

**Table 2.** Overall pairwise distance between *Aeromonas* plasmids and strains containing their closest sequence homologue after analysis with MEGA X. Query coverage and identity (%) are values from NCBI BLAST search.

	Plasmid/gene	^a^Query Coverage (%)	^a^Identity (%)	^b^Overall pairwise Distance between the two closest strains
1.	A. salmonicida pS121-1a (CP022170.1) A. salmonicida plasmid pS121-1b (MF495478.1)	100 97.00	100 99.96	7.07 ± 0.18
2.	^d^A. Hydrophila pWCX23 (CP028419) Un-named plasmid from strain 23-C-23 (CP038466.1)	100 100	100 99.99	14.44 ± 1.40
3.	^e^A. veronii pASP-a58 (CP014775.1) Un-named plasmid from Providencia stuartii strain FDAARGOS_645 (CP044075.1)	100 92	100 100	13.68 ±128
4.	^f^A. caviae pGSH8M-1-1 (AP019196.1) A. salmonicida pS44-1 (CP022176.1)	100 51	100 99.92	17.34 ± 2.7
5.	^g^A. bestiarum pAb5S9 (EF495198.1) TPA_inf:A. bestiarum strain 5S9 plasmid pAb5S9 (BK008853.1)	100 100	100 100	11.04 ± 1.23
6.	^h^A. sobria pAQ2-1 (JN315884.1) A. hydrophila pAQ2-2 (JN315885.1)	100 100	100 99.17	6.3 ± 1.6
7.	*blaCEPH-A3* ^i^A veronii B beta-lactamase cephA3 (AY112998.1) A. veronii metallo-beta-lactamase CphA3(NG_047666.1)	100 87	100 100	7.11 ± 1.63

a: analysis from NCBI; b: Analysis with MEGA X; c: 98 % (2 out of 100 sequences) of query coverage ranged from 2–56%; d : 95% of query coverage ranged from 33–94%; e: 99% of query coverage ranged from 25–92%; f: 99 % of query coverage ranged from 4–51%; g: 98% of query coverage ranged from 19–20%; h : 98 % of query coverage ranged from 6–65%; i: 93% of query coverage ranged from 61–94%.

Only 20 out of 700 sequences (including the query sequence) obtained after the BLAST activity showed a sequence query coverage of 95 % and above [See supplementary files]. It has been explained [33] that, low query coverage is when there is a poor overlap between the query sequence and sequences in GenBank. This normally indicates that the query reference or the sequences in the NCBI nucleotide collections are too short. It could also mean that the query reference is a unique sequence. The high variation of per cent query coverage observed may lead to erroneous comparisons, hence, the mean pairwise distance between sequences was estimated without alignment for just the top two BLAST hits for each plasmid/gene using MEGA X. The poor query coverage overall made the data unsuitable for classical multiple sequence alignment and the argument of others on the benefits of alignment-free calculations [91]–[93] was relied upon to calculate the mean distances. Unsurprisingly, it was found that the pair of sequences from the same genus and species had lower pairwise distances whereas it was higher for sequences of pairs from the different genus (Table 2). Contrastingly, the lowest mean pairwise distance of 6.3 ± 1.6 observed for sequences from *A. sobria* pAQ2-1 (JN315884.1) and *A. hydrophila* pAQ2-2 (JN315885.1) suggested closer historical connections. Both plasmids were isolated from the same source and they are assumed to be the same plasmid [94].

## 4. Conclusions

The *in silico* analysis of *Aeromonas* plasmids for acquired AMR genes carried out showed that there was no positive linear correlation between size and GC content for the *Aeromonas* plasmid set analyzed. This allows the conclusion that plasmid size was not dependent on GC content and vice versa. Some plasmids appear to be quite good in inter-genus AMR gene acquisition from other Gram-negative organisms. Broadly translated, this finding indicates that *Aeromonas* plasmids are prolific agents for the spread of AMR genes. The *blaCEPH-A3* gene appears to be orthologous and it may be beneficial for public health if it is subjected to increased surveillance. The low query coverage for homologs of plasmid sequences with high AMR gene prevalence found in this study suggests unique plasmid sequences that are not widely spread in other species. The main conclusion that can be drawn from this is that the presence of AMR genes may not necessarily translate to strong phenotypic expression. Hence, it will be important that future researchers perform further analysis to validate the phenotypic presence of the AMR genes detected. Overall, the findings in this study emphasizes that performing *in silico* studies is informative and can give an overview of AMR acquisition.
