# Supplementary material for: An in silico analysis of acquired antimicrobial resistance genes in Aeromonas plasmids
Source: AIMS Microbiol. 2020 Mar 16;6(1):75–91. doi: 10.3934/microbiol.2020005 (PMC7099201; doi:10.3934/microbiol.2020005)
Supplement: Supplementary file 5 [file microbiol-06-01-005-s005.pdf]

BLAST ® » blastn suite » results for RID-VGDG5G4K015

Job Title	gb|CP022170.1| ...
RID	VGDG5G4K015 Search expires on 10-30 20:52 pm
Program	BLASTN
Database	nt
Query ID	CP022170.1
Description	Aeromonas salmonicida strain S121 plasmid pS121-1a, complete sequence ...
Molecule type	nucleic acid
Query Length	195805

Descriptions

Description	Max Score	Total Score	Query Cover	E value	Per. Ident	Accession
Aeromonas salmonicida strain S121 plasmid pS121-1a, complete sequence	3.616e+05	4.119e+05	100%	0.0	100.00%	CP022170.1
Aeromonas salmonicida plasmid pS121-1b, complete sequence	1.316e+05	3.986e+05	97%	0.0	99.96%	MF495478.1
Aeromonas sp. ASNIH4 plasmid pAER-f909, complete sequence	24399	1.538e+05	56%	0.0	90.16%	CP026221.1
Pseudomonas aeruginosa plasmid pA681-IMP, complete sequence	15836	68066	15%	0.0	99.95%	MF344570.1
Pseudomonas putida strain SY153 plasmid pSY153-MDR, complete sequence	15836	91040	16%	0.0	99.95%	KY883660.1
Pseudomonas aeruginosa strain PA121617 plasmid pBM413, complete sequence	15836	68492	15%	0.0	99.95%	CP016215.1
Pseudomonas aeruginosa plasmid p727-IMP, complete sequence	15387	53055	13%	0.0	99.01%	MF344568.1
Pseudomonas aeruginosa strain AR_0111 chromosome, complete genome	15317	71319	14%	0.0	99.98%	CP032257.1
Pseudomonas aeruginosa strain AR_0230 chromosome, complete genome	15317	65884	13%	0.0	99.98%	CP027174.1
Pseudomonas aeruginosa DNA, complete genome, strain: NCGM257	15317	77927	14%	0.0	99.98%	AP014651.1
Aeromonas caviae strain WCW1-2 chromosome, complete genome	12988	1.631e+05	22%	0.0	97.81%	CP039832.1
Pseudomonas aeruginosa strain CCUG 51971 chromosome, complete genome	12864	1.091e+05	15%	0.0	99.07%	CP043328.1
Pseudomonas putida strain PP112420, complete genome	12848	67795	13%	0.0	99.87%	CP017073.1
Aeromonas caviae GSH8M-1 plasmid pGSH8M-1-2 DNA, complete genome	12746	21590	5%	0.0	99.90%	AP019197.1
Pseudomonas rhodesiae strain BS2777 genome assembly, chromosome: I	12718	28730	9%	0.0	99.84%	LT629801.1
Pseudomonas aeruginosa plasmid Rms149	12706	25651	8%	0.0	99.80%	AJ877225.1
Klebsiella pneumoniae strain KPNIH48 plasmid pKPN-10f7, complete sequence	12683	15879	5%	0.0	99.70%	CP026397.1
Klebsiella pneumoniae strain KPNIH48 plasmid pKPN-8c6e, complete sequence	12682	15941	4%	0.0	99.70%	CP026396.1
Pseudomonas veronii 1YdBTEX2 genome assembly, chromosome: PVE_r2	12652	29533	6%	0.0	99.63%	LT599584.1
Aeromonas sp. ASNIH2 chromosome, complete genome	12650	96511	10%	0.0	99.60%	CP026406.1
Pseudomonas aeruginosa strain PA34 plasmid pMKPA34-1, complete sequence	12397	48268	15%	0.0	99.76%	MH547560.1
Pseudomonas sp. BJP69 chromosome, complete genome	12244	46185	13%	0.0	98.28%	CP041933.1
Klebsiella pneumoniae strain KP18-29 plasmid p18-29-MDR, complete sequence	12039	59906	13%	0.0	97.78%	MK262712.1

Description	Max Score	Total Score	Query Cover	E value	Per. Ident	Accession
Stenotrophomonas maltophilia strain NCTC10259 genome assembly, chromosome: 1	11958	18480	6%	0.0	92.92%	LR134324.1
Stenotrophomonas sp. pho chromosome	11952	18521	6%	0.0	92.91%	CP029759.1
Stenotrophomonas sp. PAMC25021 chromosome, complete genome	11952	18475	6%	0.0	92.91%	CP039255.1
Stenotrophomonas maltophilia strain CSM2 chromosome, complete genome	11952	17484	5%	0.0	92.91%	CP025298.1
Pseudomonas putida S16, complete genome	11952	18288	6%	0.0	92.91%	CP002870.1
Pseudomonas putida strain 12969 plasmid p12969-DIM, complete sequence	11937	66639	14%	0.0	97.52%	KU130294.1
Transposon Tn501 from Pseudomonas aeruginosa plasmid pVS1 encoding mercuric ion resistance determinant. Genes: merR (regulation), merT and merP (transport), merA (reductase), merD (not known); two open reading frames of unknown function (possibly one is merE); res site, tnpR and tnpA (transposition)	11936	17659	5%	0.0	92.89%	Z00027.1
Shigella flexneri 5a plasmid virulence plasmid pWR501, complete sequence	11930	17654	5%	0.0	92.87%	AF348706.1
Aeromonas hydrophila subsp. hydrophila strain WCHAH045096 chromosome, complete genome	11904	1.215e+05	15%	0.0	95.21%	CP028568.2
Bacterium 72B plasmid pTOR_01, complete sequence	11806	15198	4%	0.0	99.98%	JX843237.1
Pseudomonas aeruginosa strain 1334/14 chromosome, complete genome	11701	91151	16%	0.0	97.94%	CP035739.1
Uncultured bacterium plasmid pKAZ5, complete sequence	11677	24815	6%	0.0	99.02%	KR827394.1
Salmonella enterica subsp. enterica serovar Rissen plasmid pSR166 RepA plasmid replication protein (repA) gene, partial cds; RepF regulator protein (repF), ParA partitioning protein (parA), and invertase/recombinase genes, complete cds; class 1 integron, complete sequence; and hypothetical protein genes, complete cds	11206	23279	6%	0.0	99.93%	KU886277.1
Pseudomonas sp. HLS-6 chromosome, complete genome	11110	27166	7%	0.0	96.36%	CP024478.1
Pseudomonas aeruginosa strain Y31 chromosome, complete genome	10986	28594	10%	0.0	99.87%	CP030910.1
Pseudomonas putida strain JBC17 chromosome, complete genome	10986	10986	3%	0.0	99.87%	CP029693.1
Pseudomonas aeruginosa isolate paerg005 genome assembly, chromosome: 0	10973	43078	12%	0.0	99.83%	LR130534.1
Pseudomonas aeruginosa isolate paerg009 genome assembly, chromosome: 0	10973	43078	12%	0.0	99.83%	LR130533.1
Pseudomonas sp. PONIH3 chromosome, complete genome	10973	32296	7%	0.0	99.83%	CP026386.1
Pseudomonas mandelii JR-1 plasmid, complete sequence	10960	17477	5%	0.0	99.77%	CP005961.1
Pseudomonas mendocina strain MAE1-K chromosome, complete genome	10949	27742	5%	0.0	99.75%	CP023641.1
Escherichia coli strain HS30-1 plasmid pHS30-1, complete sequence	10940	50650	8%	0.0	99.95%	CP029493.1
Escherichia coli strain EP28 plasmid pHNEP28_cfr, complete sequence	10938	26083	3%	0.0	99.95%	KT845955.1
Pseudomonas sp. strain FFUP_PS_41 plasmid pJBCL41, complete sequence	10936	43621	12%	0.0	99.68%	MK496050.1
Pseudomonas putida plasmid pDK1 DNA, complete sequence, strain: HS1	10936	13659	3%	0.0	99.65%	AB434906.1
Escherichia coli GSH8M-2 plasmid pGSH8M-2-2 DNA, complete genome	10935	22012	4%	0.0	99.93%	AP019677.1

Description	Max Score	Total Score	Query Cover	E value	Per. Ident	Accession
Pseudomonas aeruginosa plasmid pR31014-IMP, complete sequence	10935	67391	15%	0.0	99.68%	MF344571.1
Pseudomonas sp. LM13 transposon Tn4662a, complete sequence	10925	13186	3%	0.0	99.65%	KJ920396.1
Pseudomonas oleovorans strain T9AD genome assembly, chromosome: POT9AD	10920	16369	6%	0.0	90.65%	LR130779.1
Escherichia coli strain 1079 plasmid p1079-IncFIB-N, complete sequence	10900	29845	9%	0.0	99.83%	MG825383.1
Aeromonas hydrophila strain 23-C-23 plasmid unnamed, complete sequence	10870	61808	17%	0.0	100.00%	CP038466.1
Aeromonas hydrophila strain WCX23 plasmid unnamed, complete sequence	10870	61961	17%	0.0	100.00%	CP038464.1
Aeromonas hydrophila strain WCX23 plasmid pWCX23_1, complete sequence	10870	61961	17%	0.0	100.00%	CP028419.1
Vibrio alginolyticus strain VAS3-1 plasmid pVAS3-1, complete sequence	10870	68133	14%	0.0	100.00%	KU160531.1
Aeromonas hydrophila strain ZYAH75 chromosome, complete genome	10864	67883	16%	0.0	100.00%	CP016990.1
Citrobacter freundii strain 18-1 plasmid pBKPC18-1, complete sequence	10706	20070	7%	0.0	96.96%	CP022275.1
Pseudomonas aeruginosa strain AR_0110 chromosome, complete genome	10643	45204	10%	0.0	100.00%	CP029745.1
Pseudomonas putida strain DLL-E4, complete genome	10318	55775	11%	0.0	99.73%	CP007620.1
Pseudomonas mendocina strain 57 plasmid pAER57, complete sequence	10316	27938	9%	0.0	99.72%	MK671726.1
Pseudomonas monteilii strain B5 plasmid pSH5-1, complete sequence	10316	15650	5%	0.0	99.72%	CP022563.1
Pseudomonas aeruginosa genome assembly PAMH19, plasmid pPAMH19, plasmid : pPAMH19	10316	18751	5%	0.0	99.72%	LN809998.1
Stenotrophomonas rhizophila strain GA1 plasmid unnamed3, complete sequence	10310	14198	5%	0.0	99.70%	CP031732.1
Pseudomonas aeruginosa strain 1160 plasmid p1160-VIM, complete sequence	10309	48762	11%	0.0	99.68%	MF144194.2
Pseudomonas veronii strain Pvy plasmid unnamed, complete sequence	10303	20617	6%	0.0	99.68%	CP039632.1
Pseudomonas aeruginosa isolate RW109 genome assembly, chromosome: Main_chromosome	10301	36411	9%	0.0	99.68%	LT969520.1
Pseudomonas sp. XWY-1 plasmid, complete genome	10257	54221	10%	0.0	99.56%	CP026333.1
Pseudomonas sp. strain ANT_H62 plasmid pA62H2, complete sequence	10250	10397	2%	0.0	99.54%	MK376351.1
Pseudomonas putida ND6 plasmid pND6-1, complete sequence	10250	10383	2%	0.0	99.54%	AY208917.2
Pseudomonas putida HB3267 plasmid pPC9, complete sequence	10248	72967	12%	0.0	99.54%	CP003739.1
Pseudomonas aeruginosa strain PA83 plasmid unnamed1, complete sequence	9736	27688	7%	0.0	92.03%	CP017294.1
P.putida plasmid pPGH1 DNA, tnpR and tnpA genes	9683	9683	2%	0.0	97.84%	Y09450.1
Pseudomonas cerasi isolate PL963 genome assembly, plasmid: PP1	9247	12736	3%	0.0	99.88%	LT963396.1
Klebsiella oxytoca strain AR_0028 plasmid unitig_2_pilon, complete sequence	9215	16617	5%	0.0	90.71%	CP026717.1

Description	Max Score	Total Score	Query Cover	E value	Per. Ident	Accession
Aeromonas caviae strain F120AE51 truncated integrase (intI1), aminoglycoside-2'-adenyltransferase (aadB), quinolone-resistance protein QnrVC4 (qnrVC4), aminoglycoside (6')-N-acetyltransferase (aacA4), chloramphenicol resistance protein CmlA5 (cmlA5), class D beta-lactamase OXA-10 (blaOXA-10), aminoglycoside 3'-adenyltransferase (aadA1), extended-spectrum class A beta-lactamase VEB-1 (blaVEB-1), and aminoglycoside-2'-adenyltransferase (aadB) genes, complete cds; insertion sequence, complete sequence; disrupted tniR gene, partial sequence; and TniQ protein (tniQ) and TniB protein (tniB) genes, complete cds	8789	11796	3%	0.0	99.98%	KU886278.1
Enterobacter cloacae complex 'Hoffmann cluster III' integron:class 1 In1374 DNA, complete sequence, strain: AZ 886	8641	20970	5%	0.0	99.94%	LC224312.1
Pseudomonas aeruginosa strain S86968, complete genome	8497	44493	10%	0.0	99.63%	CP008865.2
Aeromonas hydrophila subsp. hydrophila strain WCHAH045096 plasmid p1_045096, complete sequence	8486	8486	2%	0.0	99.46%	CP028562.2
Aeromonas hydrophila subsp. hydrophila strain WCHAH045096 plasmid p2_045096, complete sequence	8486	12084	5%	0.0	99.32%	CP028563.1
Achromobacter xylosoxidans strain X02736 clone fosmid AMO9, partial sequence	8469	26411	9%	0.0	99.40%	JX448550.1
Pseudomonas aeruginosa strain K34-7 chromosome, complete genome	8468	68220	13%	0.0	99.96%	CP029707.1
Pseudomonas aeruginosa strain PA83, complete genome	8468	79387	14%	0.0	99.96%	CP017293.1
Pseudomonas aeruginosa strain GIMC5002:PAT-169 chromosome	8434	70961	13%	0.0	99.83%	CP043549.1
Pseudomonas aeruginosa strain GIMC5001:PAT-23 chromosome	8434	71202	13%	0.0	99.85%	CP043483.1
Pseudomonas aeruginosa strain FDAARGOS_571 chromosome, complete genome	8434	48628	13%	0.0	99.85%	CP033833.1
Pseudomonas aeruginosa strain AR_0357 chromosome, complete genome	8434	91809	13%	0.0	99.83%	CP027166.1
Pseudomonas aeruginosa strain E6130952, complete genome	8434	67815	11%	0.0	99.85%	CP020603.1
Pseudomonas aeruginosa DNA, complete genome, strain: NCGM 1984	8434	67722	12%	0.0	99.85%	AP014646.1
Pseudomonas aeruginosa DNA, complete genome, strain: NCGM 1900	8434	67722	12%	0.0	99.85%	AP014622.1
Pseudomonas aeruginosa strain 24Pae112 chromosome, complete genome	8432	85341	13%	0.0	99.87%	CP029605.1
Pseudomonas aeruginosa strain AR_0353 chromosome, complete genome	8431	68164	10%	0.0	99.83%	CP027172.1
Pseudomonas aeruginosa strain Pa58, complete genome	8431	88793	12%	0.0	99.85%	CP021775.1
Pseudomonas aeruginosa strain C79 genomic island sequence	8429	28397	8%	0.0	99.80%	JF826498.1
Pseudomonas aeruginosa strain 29785cz genomic sequence	8425	37924	9%	0.0	99.83%	KY860572.1
Escherichia coli strain 6409 plasmid p6409-202.186kb, complete sequence	8405	36452	11%	0.0	99.91%	CP010373.2
Pseudomonas aeruginosa strain C79 chromosome, complete genome	8395	21400	7%	0.0	99.87%	CP040684.1
Pseudomonas aeruginosa strain Pavimgi1 urocanate hydratase gene, partial cds; and VIM gene cluster, complete sequence	8379	51002	12%	0.0	99.96%	KJ463833.1
Pseudomonas aeruginosa strain PA34 chromosome, complete genome	8266	17588	7%	0.0	99.89%	CP032552.1

BLAST® » blastn suite » results for RID-VGHACATP015

Job Title	gb|CP028419.1| ...
RID	VGHACATP015 Search expires on 10-30 21:57 pm
Program	BLASTN
Database	nt
Query ID	CP028419.1
Description	Aeromonas hydrophila strain WCX23 plasmid pWCX23_1, complete sequence ...
Molecule type	nucleic acid
Query Length	165121

Descriptions

Description	Max Score	Total Score	Query Cover	E value	Per. Ident	Accession
Aeromonas hydrophila strain WCX23 plasmid pWCX23_1, complete sequence	3.049e+05	3.309e+05	100%	0.0	100.00%	CP028419.1
Aeromonas hydrophila strain 23-C-23 plasmid unnamed, complete sequence	2.710e+05	3.308e+05	100%	0.0	99.99%	CP038466.1
Aeromonas hydrophila strain WCX23 plasmid unnamed, complete sequence	2.671e+05	3.308e+05	100%	0.0	99.99%	CP038464.1
Escherichia coli O157 strain AR-0429 plasmid pAR-0429-1, complete sequence	1.788e+05	2.853e+05	90%	0.0	99.97%	CP044142.1
Salmonella enterica subsp. enterica serovar Newport str. USDA-ARS-USMARC-1928 plasmid pSNE3-1928, complete sequence	1.735e+05	2.745e+05	87%	0.0	99.98%	CP025240.1
Salmonella enterica subsp. enterica serovar Anatum str. USDA-ARS-USMARC-1736 plasmid pSAN1-1736, complete sequence	1.735e+05	3.086e+05	91%	0.0	99.98%	CP014658.1
Klebsiella pneumoniae strain Kpn642 plasmid pKP-Gr642, complete sequence	1.735e+05	2.956e+05	91%	0.0	99.98%	KR559888.1
Escherichia coli UMNK88 plasmid pUMNK88, complete sequence	1.734e+05	3.002e+05	92%	0.0	99.97%	HQ023862.1
Salmonella enterica subsp. enterica serovar Newport str. CVM 22425 plasmid pCVM22425, complete sequence	1.727e+05	3.167e+05	88%	0.0	99.98%	CP009560.1
Salmonella enterica subsp. enterica serovar Newport str. CVM N1543 plasmid pCVMN1543, complete sequence	1.727e+05	2.396e+05	66%	0.0	99.98%	CP009570.1
Salmonella enterica subsp. enterica serovar Newport str. CVM 22462 plasmid pCFSAN000934_02, complete sequence	1.727e+05	3.174e+05	88%	0.0	99.98%	CP009567.1
Salmonella enterica subsp. enterica serovar Newport str. CVM 22513 plasmid pCVM22513, complete sequence	1.727e+05	2.429e+05	66%	0.0	99.98%	CP009562.1
Salmonella enterica subsp. enterica serovar Newport strain SAP18-8729 plasmid pCFSAN074384_1, complete sequence	1.660e+05	3.169e+05	88%	0.0	99.98%	CP041209.1
Escherichia coli strain AR060302 plasmid pAR060302, complete sequence	1.609e+05	3.025e+05	91%	0.0	99.98%	FJ621588.1
Salmonella enterica subsp. enterica serovar Typhimurium str. USDA-ARS-USMARC-1896 plasmid pSTY1-1896, complete sequence	1.606e+05	2.945e+05	88%	0.0	99.92%	CP014978.1
Salmonella enterica subsp. enterica serovar Newport str. CDC 2012K-0663 plasmid pSNE2-2012K-0663, complete sequence	1.508e+05	2.096e+05	67%	0.0	99.99%	CP025245.1
Klebsiella pneumoniae strain Kpn8143 plasmid pKP-Gr8143, complete sequence	1.418e+05	2.783e+05	86%	0.0	99.99%	KR559889.1
Salmonella enterica subsp. enterica serovar Newport strain CFSAN003890 plasmid pCFSAN003890, complete sequence	1.389e+05	2.440e+05	68%	0.0	99.97%	CP016013.1
Aeromonas salmonicida subsp. salmonicida strain 2004-05MF26 plasmid pSN254b, complete sequence	1.360e+05	2.920e+05	91%	0.0	99.99%	KJ909290.1

Description	Max Score	Total Score	Query Cover	E value	Per. Ident	Accession
Salmonella enterica strain SA20025921 plasmid pSA20025921.1, complete sequence	1.360e+05	2.922e+05	91%	0.0	99.98%	CP030215.1
Vibrio cholerae strain 2012EL-2176 plasmid, complete sequence	1.360e+05	3.034e+05	91%	0.0	99.98%	CP007636.1
Escherichia coli O16:H48 strain PG20180173 plasmid pPG20180173.1-IncAC2, complete sequence	1.360e+05	3.199e+05	92%	0.0	99.98%	CP043192.1
Escherichia coli O16:H48 strain PG20180175 plasmid pPG20180175.1-IncAC2, complete sequence	1.360e+05	3.199e+05	92%	0.0	99.98%	CP043190.1
Salmonella enterica subsp. enterica serovar Heidelberg strain SL-312 plasmid pET8.1-IncAC2, complete sequence	1.360e+05	3.198e+05	92%	0.0	99.98%	CP043215.1
Proteus mirabilis strain CCUG 70746 plasmid pPmi70746_1, complete sequence	1.360e+05	2.886e+05	90%	0.0	99.98%	CP023274.1
Escherichia coli strain AMA566 plasmid pAMA566, complete sequence	1.360e+05	2.886e+05	90%	0.0	99.98%	MG450360.1
Klebsiella pneumoniae strain Kp202 plasmid pKp202_1, complete sequence	1.359e+05	3.017e+05	93%	0.0	99.98%	CP041083.1
Escherichia coli strain YDC637 plasmid pYDC637, complete sequence	1.359e+05	2.995e+05	89%	0.0	99.97%	KP056256.1
Salmonella enterica subsp. enterica serovar Heidelberg plasmid pSH111_166, complete sequence	1.359e+05	2.992e+05	91%	0.0	99.98%	JN983043.1
Providencia stuartii plasmid pMR0211, complete sequence	1.337e+05	2.887e+05	85%	0.0	99.97%	JN687470.1
Vibrio alginolyticus strain VAS3-1 plasmid pVAS3-1, complete sequence	1.300e+05	3.289e+05	96%	0.0	99.98%	KU160531.1
Salmonella enterica strain 2016K-0796 plasmid p2016K-0796, complete sequence	1.299e+05	2.710e+05	76%	0.0	99.98%	MH760469.1
Salmonella enterica subsp. enterica serovar Corvallis strain 12-01738 plasmid pSE12-01738-2, complete sequence	1.271e+05	2.851e+05	88%	0.0	99.98%	CP027679.1
Salmonella enterica subsp. enterica strain 1607:MR00001R plasmid p08-5333.1, complete sequence	1.254e+05	2.310e+05	54%	0.0	99.98%	CP039562.1
Escherichia coli strain Ecol_AZ155 plasmid pECAZ155_KPC, complete sequence	1.242e+05	3.147e+05	89%	0.0	99.88%	CP019001.1
Klebsiella pneumoniae strain AR_0139, complete genome	1.235e+05	2.036e+05	56%	0.0	99.98%	CP021960.1
Klebsiella pneumoniae strain KKp4 plasmid pKKp4-VIM, complete sequence	1.235e+05	2.832e+05	88%	0.0	99.98%	MF582638.1
Klebsiella pneumoniae strain KPN1482, complete genome	1.235e+05	2.348e+05	62%	0.0	99.97%	CP020841.1
Klebsiella pneumoniae strain T38 plasmid pT38_MCR3, complete sequence	1.234e+05	2.685e+05	82%	0.0	99.96%	MK770642.1
Klebsiella pneumoniae strain 526316 plasmid p526316-KPC, complete sequence	1.232e+05	3.211e+05	91%	0.0	99.90%	MH909327.1
Salmonella enterica subsp. enterica serovar Agona strain 44 plasmid, partial sequence	1.230e+05	3.035e+05	91%	0.0	99.98%	MK191845.1
Salmonella enterica subsp. enterica serovar Corvallis plasmid pRH-1238, complete sequence	1.227e+05	3.249e+05	93%	0.0	99.98%	KR091911.1
Salmonella enterica subsp. enterica strain YU39 plasmid pYU39_IncA/C, complete sequence	1.175e+05	2.321e+05	68%	0.0	99.97%	CP011429.1
Escherichia coli strain AMSCJX02 plasmid pAMSC1, complete sequence	1.138e+05	2.437e+05	78%	0.0	99.87%	CP031106.1
Shewanella algae strain CCU101 plasmid unnamed, complete sequence	1.137e+05	2.307e+05	77%	0.0	99.85%	CP018457.1
Klebsiella pneumoniae subsp. pneumoniae strain KpvST15_NDM plasmid pKpvST15, complete sequence	1.130e+05	2.044e+05	55%	0.0	99.94%	CP040595.1

Description	Max Score	Total Score	Query Cover	E value	Per. Ident	Accession
Salmonella enterica subsp. enterica serovar Dublin strain CVM 34981 plasmid p34981_1, complete sequence	1.115e+05	2.829e+05	88%	0.0	99.98%	CP032391.1
Salmonella enterica strain CFSAN007428 plasmid pCFSAN007428_01, complete sequence	1.114e+05	3.076e+05	92%	0.0	99.98%	CP009414.2
Salmonella enterica subsp. enterica serovar Heidelberg str. N418 plasmid pCFSAN000405_01, complete sequence	1.110e+05	3.383e+05	93%	0.0	99.99%	CP009409.2
Salmonella enterica strain AM04528 plasmid pAM04528, complete sequence	1.110e+05	3.169e+05	88%	0.0	99.99%	FJ621587.1
Salmonella enterica subsp. enterica serovar Newport str. SL254 plasmid pSN254, complete sequence	1.110e+05	3.240e+05	91%	0.0	99.99%	CP000604.1
Salmonella enterica strain CFSAN007425 plasmid pCFSAN007425_01, complete sequence	1.110e+05	3.274e+05	91%	0.0	99.99%	CP009411.2
Salmonella enterica subsp. enterica serovar Newport str. USDA-ARS-USMARC-1924 plasmid pSNE1-1924, complete sequence	1.109e+05	2.960e+05	87%	0.0	99.99%	CP025231.1
Salmonella enterica subsp. enterica serovar Newport str. USDA-ARS-USMARC-1929 plasmid pSNE1-1929, complete sequence	1.104e+05	1.850e+05	59%	0.0	99.99%	CP025242.1
Salmonella enterica subsp. enterica serovar Newport str. USDA-ARS-USMARC-1926 plasmid pSNE2-1926, complete sequence	1.104e+05	2.288e+05	67%	0.0	99.99%	CP025236.1
Escherichia coli strain Esco-36073cz plasmid pEsco-36073cz, complete sequence	1.097e+05	3.135e+05	92%	0.0	99.98%	MG252895.1
Vibrio parahaemolyticus plasmid pVPS114, complete sequence	1.088e+05	2.541e+05	83%	0.0	99.98%	KY014465.1
Salmonella enterica strain CFSAN007427 plasmid pCFSAN007427_01, complete sequence	1.085e+05	3.098e+05	91%	0.0	99.99%	CP009413.2
Providencia rettgeri strain QD51 plasmid pNDM-QD51, complete sequence	1.076e+05	2.105e+05	69%	0.0	99.94%	MH263652.1
Citrobacter freundii strain AMA332 plasmid pT1, complete sequence	1.076e+05	2.075e+05	68%	0.0	99.94%	KX147633.1
Enterobacter cloacae strain B557 plasmid pB557-NDM, complete sequence	1.076e+05	2.087e+05	69%	0.0	99.94%	KX786648.1
Klebsiella pneumoniae strain RJ119 plasmid pRJ119-NDM1, complete sequence	1.076e+05	2.828e+05	76%	0.0	99.94%	KX636095.1
Escherichia coli strain MS6198 plasmid pMS6198A, complete sequence	1.076e+05	2.087e+05	69%	0.0	99.94%	CP015835.1
Klebsiella pneumoniae strain KP1 plasmid pKP1-NDM-1, complete sequence	1.076e+05	2.109e+05	69%	0.0	99.94%	KF992018.2
Escherichia coli strain EC2 plasmid pEC2-NDM-3, complete sequence	1.076e+05	2.089e+05	69%	0.0	99.94%	KC999035.4
Providencia stuartii isolate GN576 plasmid pNDM-PstGN576, complete sequence	1.076e+05	2.076e+05	69%	0.0	99.94%	KJ802405.1
Escherichia coli isolate GN568 plasmid pNDM-EcoGN568, complete sequence	1.076e+05	2.076e+05	69%	0.0	99.94%	KJ802404.1
Escherichia coli strain N10-2337 plasmid pNDM102337, complete sequence	1.076e+05	2.107e+05	70%	0.0	99.94%	JF714412.2
Klebsiella pneumoniae strain N10-0469 plasmid pNDM10469, complete sequence	1.076e+05	2.091e+05	69%	0.0	99.94%	JN861072.1
Escherichia coli strain N10-0505 plasmid pNDM10505, complete sequence	1.076e+05	2.125e+05	70%	0.0	99.94%	JF503991.1
Escherichia coli strain K71-77 plasmid pK71-77-1-NDM, complete sequence	1.076e+05	2.122e+05	70%	0.0	99.94%	CP040884.1
Klebsiella pneumoniae strain AR_0049 plasmid unitig_1, complete sequence	1.076e+05	2.076e+05	69%	0.0	99.94%	CP018817.1
Salmonella enterica subsp. enterica serovar Stanley strain LS001 plasmid pHS36-NDM, complete sequence	1.076e+05	2.086e+05	69%	0.0	99.94%	KU726616.1

Description	Max Score	Total Score	Query Cover	E value	Per. Ident	Accession
Klebsiella pneumoniae strain ATCC BAA-2146 plasmid pNDM-US-2, complete sequence	1.076e+05	2.085e+05	69%	0.0	99.94%	KJ588779.1
Klebsiella pneumoniae strain ATCC BAA-2146 plasmid pNDM-US, complete sequence	1.076e+05	2.087e+05	69%	0.0	99.94%	CP006661.1
Citrobacter freundii strain CRE3 plasmid pCRE7-NDM, complete sequence	1.076e+05	1.942e+05	64%	0.0	99.94%	MK101346.1
Salmonella enterica subsp. enterica serovar Newport strain 0307-213, complete genome	1.073e+05	3.392e+05	70%	0.0	99.97%	CP012599.1
Vibrio alginolyticus strain Vb1796 plasmid pVb1796, complete sequence	1.066e+05	2.971e+05	91%	0.0	99.94%	MH113855.1
Salmonella enterica strain CFSAN007426 plasmid pCFSAN007426_01, complete sequence	1.063e+05	2.054e+05	63%	0.0	99.98%	CP009412.2
Escherichia coli strain AR_0069 plasmid unitig_2, complete sequence	1.060e+05	2.087e+05	69%	0.0	99.94%	CP020056.1
Escherichia coli strain APEC1990_61 plasmid pAPEC1990_61, complete sequence	1.019e+05	2.955e+05	88%	0.0	99.98%	HQ023863.1
Salmonella enterica subsp. enterica serovar Thompson strain HFCDC-SM-846 plasmid p846, complete sequence	1.008e+05	2.603e+05	75%	0.0	99.95%	CP029249.1
Klebsiella pneumoniae plasmid IncA/C-LS6, complete sequence	1.002e+05	2.931e+05	92%	0.0	99.97%	JX442976.1
Salmonella enterica subsp. enterica serovar Newport str. CVM 21538 plasmid pCVM21538, complete sequence	99542	1.686e+05	49%	0.0	99.95%	CP009563.1
Salmonella enterica subsp. enterica serovar Thompson strain SH11G0791 plasmid pSH11G0791, complete sequence	99417	2.623e+05	75%	0.0	99.95%	CP041172.1
Salmonella enterica subsp. enterica serovar Lomita strain SL131 plasmid pSL131_IncA/C-IncX3, complete sequence	98564	3.311e+05	95%	0.0	99.90%	MH105050.1
Salmonella enterica subsp. enterica serovar Newport str. USDA-ARS-USMARC-1923 plasmid pSNE2-1923	98553	2.473e+05	64%	0.0	99.97%	CP025275.1
Salmonella enterica subsp. enterica serovar Newport str. CVM 21550 plasmid pCVM21550, complete sequence	98499	2.429e+05	66%	0.0	99.95%	CP009564.1
Salmonella enterica subsp. enterica strain SA972816 plasmid p972816 sequence	97109	1.004e+05	33%	0.0	99.97%	CP007487.1
Klebsiella pneumoniae strain WCHKP7E2 plasmid pCMY2_085072, complete sequence	96119	3.625e+05	93%	0.0	99.67%	CP028804.2
Klebsiella pneumoniae strain N201205880 plasmid p205880-Ct1/2, complete sequence	96119	2.788e+05	87%	0.0	99.67%	MF344573.1
Proteus mirabilis strain Pm14C18 plasmid pPm14C18, complete sequence	96119	3.155e+05	91%	0.0	99.67%	KU605240.1
Escherichia coli strain ECCWS199 plasmid pTB221, complete sequence	96113	3.048e+05	96%	0.0	99.67%	CP032238.1
Escherichia coli strain cq9 plasmid unnamed3, complete sequence	96108	3.329e+05	94%	0.0	99.67%	CP031549.1
Salmonella enterica subsp. enterica serovar Typhimurium plasmid pYT3 DNA, complete sequence	91532	1.876e+05	61%	0.0	99.98%	AB591424.1
Escherichia coli strain 190 plasmid unnamed1, complete sequence	91245	2.498e+05	80%	0.0	99.97%	CP020524.1
Serratia marcescens strain M17468 plasmid pSMA17468, complete sequence	90485	2.086e+05	69%	0.0	99.93%	MK123268.1
Escherichia coli strain M17386 plasmid pECO17386, complete sequence	90485	2.087e+05	69%	0.0	99.93%	MK123267.1
Enterobacter cloacae strain ECL17464 plasmid pECL17464, complete sequence	90485	2.108e+05	69%	0.0	99.93%	MH995508.1
Citrobacter amalonaticus strain M21015 plasmid pNDM-M21015, complete sequence	90485	2.087e+05	69%	0.0	99.93%	MK041212.1

BLAST ® » **blastn suite** » results for RID-VGHH2SFU014

Job Title	CP014775:Aeromonas veronii strain AVNIH1 plasmid... ...
RID	VGHH2SFU014 Search expires on 10-30 22:01 pm
Program	BLASTN
Database	nt
Query ID	CP014775.1
Description	Aeromonas veronii strain AVNIH1 plasmid pASP-a58, complete sequence ...
Molecule type	nucleic acid
Query Length	198307

Descriptions

Description	Max Score	Total Score	Query Cover	E value	Per. Ident	Accession
Aeromonas veronii strain AVNIH1 plasmid pASP-a58, complete sequence	3.662e+05	4.536e+05	100%	0.0	100.00%	CP014775.1
Providencia stuartii strain FDAARGOS_645 plasmid unnamed1, complete sequence	1.172e+05	3.960e+05	92%	0.0	100.00%	CP044075.1
Proteus mirabilis strain AR_0156 plasmid unitig_1, complete sequence	1.133e+05	3.472e+05	81%	0.0	100.00%	CP021853.1
Escherichia coli strain Ec78 plasmid pEc78, complete sequence	1.052e+05	3.122e+05	73%	0.0	99.98%	KY887595.1
Escherichia coli strain Ec19 plasmid pEc19, complete sequence	1.052e+05	3.075e+05	77%	0.0	99.97%	KY887591.1
Citrobacter freundii strain Cf53 plasmid pCf53, complete sequence	1.052e+05	3.775e+05	85%	0.0	99.97%	KY887593.1
Citrobacter freundii strain Cf52 plasmid pCf52, complete sequence	1.052e+05	4.073e+05	85%	0.0	99.97%	KY887592.1
Escherichia coli strain Ec9 plasmid pEc9, complete sequence	1.052e+05	4.056e+05	78%	0.0	99.96%	KY887590.1
Escherichia coli strain K-12 plasmid R16a, complete sequence	99046	2.529e+05	66%	0.0	99.93%	KX156773.1
Providencia stuartii strain BE2467 isolate CAUTI plasmid pPS1, complete sequence	99029	2.328e+05	64%	0.0	99.92%	CP017055.1
Klebsiella pneumoniae isolate 833f714a-b38d-11e9-8998-68b599768938 genome assembly, plasmid: p13ARS_VSM0593-1	98228	3.305e+05	81%	0.0	99.97%	LR697125.1
Klebsiella pneumoniae plasmid pHM881QN DNA, complete sequence, strain: Y881	94747	3.052e+05	79%	0.0	99.99%	LC055503.1
Citrobacter freundii strain JY-17 plasmid pCFJY-17, complete sequence	93461	3.290e+05	83%	0.0	99.99%	MH763829.1
Escherichia coli strain Ecol_732 plasmid pEC732_IMP14, complete sequence	93066	2.983e+05	72%	0.0	99.96%	CP015139.1
Aeromonas hydrophila plasmid pR148, complete sequence	93066	2.789e+05	74%	0.0	99.96%	JX141473.1
Escherichia coli strain EC17GD31 plasmid pGD31-NDM, complete sequence	93061	2.837e+05	71%	0.0	99.95%	CP031297.1
Klebsiella pneumoniae plasmid pRMH760, complete sequence	93061	3.021e+05	74%	0.0	99.95%	KF976462.2
Uncultured bacterium plasmid pKAZ4, complete sequence	93059	2.954e+05	77%	0.0	99.95%	KR827393.1
Klebsiella pneumoniae strain AR_0076 plasmid unnamed1, complete sequence	93055	3.121e+05	78%	0.0	99.95%	CP032168.1
Klebsiella quasipneumoniae strain CAV2018 plasmid pKPC_CAV2018-435, complete sequence	93055	2.354e+05	59%	0.0	99.95%	CP029431.1
Klebsiella quasipneumoniae strain CAV1947 plasmid pKPC_CAV1947-412, complete sequence	93055	2.228e+05	57%	0.0	99.95%	CP029442.1
Vibrio alginolyticus strain Vb1394 plasmid pC1394, complete sequence	93053	2.824e+05	73%	0.0	99.95%	MH457126.1

Description	Max Score	Total Score	Query Cover	E value	Per. Ident	Accession
Escherichia coli strain N15-01078 plasmid pNDM15-1078, complete sequence	93050	3.096e+05	74%	0.0	99.95%	CP012902.1
Salmonella enterica subsp. enterica serovar Montevideo str. CDC 2010K-0257 plasmid pSMO-2010K-0257, complete sequence	93048	2.159e+05	59%	0.0	99.95%	CP020913.1
Escherichia coli strain VA292 plasmid pDGO100, complete sequence	93044	3.275e+05	74%	0.0	99.95%	KU997026.1
Enterobacter hormaechei strain S5 plasmid pIncAC2-1502262, complete sequence	93035	3.135e+05	78%	0.0	99.95%	CP031573.1
Enterobacter hormaechei strain S6 plasmid pIncAC2-1502264, complete sequence	93024	3.166e+05	78%	0.0	99.94%	CP031576.1
Klebsiella pneumoniae strain S12 plasmid pIncAC2-1502320, complete sequence	93009	3.134e+05	78%	0.0	99.94%	CP031584.1
Enterobacter hormaechei strain S13 plasmid pIncAC2-1301491, complete sequence	92978	3.120e+05	78%	0.0	99.93%	CP031570.1
Escherichia coli strain S10 plasmid pIncAC2-1502318, complete sequence	92957	3.165e+05	78%	0.0	99.92%	CP031610.1
Klebsiella pneumoniae isolate 8329a5f4-b38d-11e9-8998-68b599768938 genome assembly, plasmid: p13ARS_GMH0099	92246	4.906e+05	85%	0.0	99.99%	LR697099.1
Klebsiella pneumoniae strain KP36 plasmid 2, complete sequence	88684	2.618e+05	59%	0.0	99.96%	CP017387.1
Escherichia coli plasmid pM216_AC2 DNA, complete genome, isolate: M216	87329	2.981e+05	76%	0.0	99.99%	AP018145.1
Proteus mirabilis strain CCUG 70746 plasmid pPmi70746_1, complete sequence	87227	3.462e+05	76%	0.0	99.96%	CP023274.1
Salmonella enterica subsp. enterica serovar Heidelberg plasmid pSH111_166, complete sequence	87179	2.986e+05	72%	0.0	99.94%	JN983043.1
Salmonella enterica subsp. enterica strain SA972816 plasmid p972816 sequence	87174	93033	26%	0.0	99.94%	CP007487.1
Escherichia coli O157 strain AR-0429 plasmid pAR-0429-1, complete sequence	87166	2.865e+05	74%	0.0	99.94%	CP044142.1
Salmonella enterica subsp. enterica serovar Newport strain SAP18-8729 plasmid pCFSAN074384_1, complete sequence	87166	2.989e+05	69%	0.0	99.94%	CP041209.1
Salmonella enterica subsp. enterica serovar Newport strain 0307-213, complete genome	87166	4.172e+05	68%	0.0	99.94%	CP012599.1
Salmonella enterica subsp. enterica serovar Newport str. CVM 22513 plasmid pCVM22513, complete sequence	87166	2.274e+05	51%	0.0	99.94%	CP009562.1
Salmonella enterica subsp. enterica serovar Newport str. CVM 22425 plasmid pCVM22425, complete sequence	87166	3.007e+05	69%	0.0	99.94%	CP009560.1
Salmonella enterica subsp. enterica serovar Newport str. CVM N1543 plasmid pCVMN1543, complete sequence	87166	2.273e+05	51%	0.0	99.94%	CP009570.1
Salmonella enterica subsp. enterica serovar Newport str. CVM 22462 plasmid pCFSAN000934_02, complete sequence	87166	3.014e+05	69%	0.0	99.94%	CP009567.1
Salmonella enterica subsp. enterica serovar Newport str. USDA-ARS-USMARC-1923 plasmid pSNE2-1923	87161	1.995e+05	46%	0.0	99.94%	CP025275.1
Aeromonas hydrophila strain 23-C-23 plasmid unnamed, complete sequence	87131	3.117e+05	75%	0.0	99.92%	CP038466.1
Aeromonas hydrophila strain WCX23 plasmid unnamed, complete sequence	87131	3.117e+05	75%	0.0	99.92%	CP038464.1
Aeromonas hydrophila strain WCX23 plasmid pWCX23_1, complete sequence	87118	2.859e+05	73%	0.0	99.92%	CP028419.1
Salmonella enterica subsp. enterica serovar Lomita strain SL131 plasmid pSL131_IncA/C-IncX3, complete sequence	87116	2.896e+05	76%	0.0	99.92%	MH105050.1
Salmonella enterica subsp. enterica serovar Thompson strain SH11G0791 plasmid pSH11G0791, complete sequence	87114	2.815e+05	62%	0.0	99.92%	CP041172.1

Description	Max Score	Total Score	Query Cover	E value	Per. Ident	Accession
Salmonella enterica subsp. enterica serovar Thompson strain HFCDC-SM-846 plasmid p846, complete sequence	87114	2.187e+05	59%	0.0	99.92%	CP029249.1
Salmonella enterica subsp. enterica serovar Newport str. CVM 21550 plasmid pCVM21550, complete sequence	87109	2.273e+05	51%	0.0	99.92%	CP009564.1
Salmonella enterica subsp. enterica serovar Newport str. CVM 21538 plasmid pCVM21538, complete sequence	87109	1.696e+05	38%	0.0	99.92%	CP009563.1
Salmonella enterica subsp. enterica serovar Typhi strain 80-2002 genome assembly, plasmid: 2	86104	4.161e+05	80%	0.0	99.96%	LT904892.1
Citrobacter freundii strain MRSN11938 plasmid pMRVIM0912, complete sequence	85454	2.663e+05	72%	0.0	99.94%	KP975074.1
Klebsiella quasipneumoniae strain CAV2013 plasmid pKPC_CAV2013, complete sequence	85006	2.383e+05	58%	0.0	99.95%	CP029436.1
Klebsiella pneumoniae plasmid IncA/C-LS6, complete sequence	84710	2.968e+05	75%	0.0	99.97%	JX442976.1
Salmonella enterica subsp. enterica serovar Agona strain 44 plasmid, partial sequence	84169	3.060e+05	75%	0.0	99.95%	MK191845.1
Escherichia coli strain 165 plasmid unnamed4, complete sequence	83345	93707	25%	0.0	99.92%	CP020513.1
Acinetobacter baumannii strain PB364 chromosome, complete genome	83157	1.262e+05	29%	0.0	100.00%	CP040425.1
Klebsiella pneumoniae strain 526316 plasmid p526316-KPC, complete sequence	83139	4.037e+05	76%	0.0	99.92%	MH909327.1
Escherichia coli strain Ecol_AZ155 plasmid pECAZ155_KPC, complete sequence	83133	2.872e+05	73%	0.0	99.92%	CP019001.1
Salmonella enterica subsp. enterica serovar Typhimurium str. CDC 2010K-1587 strain USDA-ARS-USMARC-1908 plasmid pSTY1-2010K-1587, complete sequence	82409	1.493e+05	41%	0.0	99.85%	CP016864.1
Salmonella enterica subsp. enterica serovar Typhimurium var. 5- str. CFSAN001921 plasmid unnamed, complete sequence	82409	2.415e+05	65%	0.0	99.85%	CP006050.1
Salmonella enterica subsp. enterica serovar Heidelberg strain 5 plasmid p3, complete sequence	82402	1.663e+05	45%	0.0	99.85%	CP031362.1
Escherichia coli J53 plasmid pMG252, complete sequence	82053	3.070e+05	74%	0.0	99.96%	MK638972.1
Citrobacter freundii complex sp. CFNIH4 plasmid pCFR-0b27, complete sequence	82047	3.109e+05	75%	0.0	99.96%	CP026233.1
Pantoea sp. PSNIH2 plasmid pPSP-100, complete sequence	82031	2.597e+05	72%	0.0	99.95%	CP009868.1
Escherichia coli strain J53 plasmid pMG252A, complete sequence	82003	3.380e+05	74%	0.0	99.94%	MK733575.1
Vibrio parahaemolyticus plasmid pVPS114, complete sequence	81643	2.257e+05	61%	0.0	99.94%	KY014465.1
Klebsiella pneumoniae strain CRE114 plasmid pIMP-PH114, complete sequence	81028	2.850e+05	75%	0.0	99.99%	KF250428.1
Klebsiella pneumoniae strain JS187 plasmid p187-4, complete sequence	80718	1.704e+05	44%	0.0	99.99%	CP025470.1
Klebsiella pneumoniae isolate 97ca48c2-b809-11e8-aae5-3c4a9275d6c8 genome assembly, chromosome: 1	80708	1.651e+05	46%	0.0	99.99%	LR596810.1
Proteus mirabilis strain T21 plasmid pT212, complete sequence	80707	2.926e+05	73%	0.0	99.98%	CP017084.1
Klebsiella pneumoniae strain TVGHCRE225 plasmid unnamed2, complete sequence	80675	2.848e+05	73%	0.0	99.97%	CP023724.1
Escherichia coli strain 513 genome assembly, plasmid: RCS30_p	80675	2.458e+05	68%	0.0	99.97%	LT985224.1
Proteus mirabilis strain A64421 plasmid pPM64421a, complete sequence	80675	2.727e+05	73%	0.0	99.97%	MF150118.1

Description	Max Score	Total Score	Query Cover	E value	Per. Ident	Accession
Klebsiella pneumoniae strain 397108 plasmid p397108-Ct2, complete sequence	80274	1.620e+05	42%	0.0	99.99%	MH917284.1
Klebsiella pneumoniae strain AR_0079 plasmid unnamed5, complete sequence	79624	2.728e+05	73%	0.0	99.97%	CP028996.1
Escherichia coli strain K-12 plasmid IP40a, complete sequence	77294	2.528e+05	66%	0.0	99.95%	KX156772.1
Pseudomonas aeruginosa strain IP40a plasmid pIP40a, complete sequence	77277	2.529e+05	66%	0.0	99.94%	KX709966.1
Escherichia coli strain 548 genome assembly, plasmid: RCS24TR548_p	76631	2.890e+05	74%	0.0	99.96%	LT985222.1
Escherichia coli strain 83 genome assembly, plasmid: RCS1TR83_p	76631	2.881e+05	74%	0.0	99.96%	LT985220.1
Escherichia coli strain 89 genome assembly, plasmid: RCS2TR89_p	76624	2.905e+05	74%	0.0	99.96%	LT985225.1
Providencia stuartii strain FDAARGOS_87 plasmid unnamed1, complete sequence	76561	2.555e+05	72%	0.0	99.93%	CP031512.1
Klebsiella pneumoniae strain Klebsiella pneumoniae KLPN57 genome assembly, plasmid: I	76561	4.048e+05	77%	0.0	99.93%	LT882698.1
Klebsiella aerogenes strain G7 plasmid pGPN1, complete sequence	76561	2.880e+05	74%	0.0	99.93%	CP011540.1
Enterobacter cloacae isolate Enterobacter cloacae ENCL58 genome assembly, plasmid: I	76550	2.791e+05	73%	0.0	99.93%	LT882699.1
Vibrio cholerae strain ICDC-1447 plasmid pVC1447, complete sequence	75582	2.992e+05	74%	0.0	99.99%	KM083064.1
Providencia rettgeri strain AR_0082 plasmid unnamed, complete sequence	75074	2.545e+05	70%	0.0	99.99%	CP029737.1
Salmonella enterica strain CFSAN064034 plasmid pGMI17-002_1, complete sequence	75069	3.249e+05	77%	0.0	99.99%	CP028170.1
Klebsiella pneumoniae strain Kp55 plasmid pKp55, complete sequence	74434	3.638e+05	84%	0.0	99.97%	KY887594.1
Providencia stuartii plasmid pTC2, complete sequence	74169	3.552e+05	77%	0.0	99.96%	JQ824049.1
Photobacterium damselae subsp. piscicida plasmid pP91278 DNA, complete sequence	73844	2.575e+05	64%	0.0	99.92%	AB277724.1
Vibrio parahaemolyticus plasmid pVPS129, complete sequence	73776	2.394e+05	69%	0.0	98.71%	KY014464.1
Citrobacter freundii strain 164 plasmid pCf164_LMB-1, complete sequence	73015	2.613e+05	69%	0.0	99.29%	MH475146.1
Klebsiella pneumoniae strain 1_GR_13 plasmid IncAC2, complete sequence	72882	2.629e+05	69%	0.0	99.96%	CP027043.1
Klebsiella pneumoniae strain 2_GR_12 plasmid IncAC2	72882	2.523e+05	67%	0.0	99.96%	CP027055.1
Klebsiella pneumoniae strain 16_GR_13 plasmid IncAC2, complete sequence	72882	2.214e+05	62%	0.0	99.96%	CP027038.1
Escherichia coli strain AMSCJX02 plasmid pAMSC1, complete sequence	72855	2.401e+05	63%	0.0	99.95%	CP031106.1
Escherichia coli strain C600_pConj125k plasmid pConj125k, complete sequence	72849	2.022e+05	40%	0.0	99.95%	MK033499.1

Graphic Summary

BLAST® » blastn suite » results for RID-VGJ5HX4V015

Job Title	AP019196:Aeromonas caviae GSH8M-1 plasmid... ...
RID	VGJ5HX4V015 Search expires on 10-30 22:12 pm
Program	BLASTN
Database	nt
Query ID	AP019196.1
Description	Aeromonas caviae GSH8M-1 plasmid pGSH8M-1-1 DNA, complete genome ...
Molecule type	nucleic acid
Query Length	153814

Descriptions

Description	Max Score	Total Score	Query Cover	E value	Per. Ident	Accession
Aeromonas caviae GSH8M-1 plasmid pGSH8M-1-1 DNA, complete genome	2.840e+05	3.399e+05	100%	0.0	100.00%	AP019196.1
Aeromonas salmonicida strain S44 plasmid pS44-1, complete sequence	31309	1.463e+05	51%	0.0	99.92%	CP022176.1
Aeromonas salmonicida subsp. salmonicida strain JF2267 plasmid pAsa4c, complete sequence	29724	1.212e+05	48%	0.0	99.93%	KT033470.1
Aeromonas salmonicida subsp. salmonicida strain 01-B522 plasmid pAsa4b, complete sequence	26242	1.116e+05	46%	0.0	99.13%	KT033469.1
Aeromonas salmonicida subsp. salmonicida A449 plasmid 4, complete sequence	26229	1.182e+05	47%	0.0	99.12%	CP000645.1
Aeromonas sp. ASNIH1 chromosome, complete genome	16297	1.640e+05	23%	0.0	99.76%	CP026228.1
Enterobacter hormaechei strain EB_P9_L5_03.19 plasmid pIMPIncH12_331kb, complete sequence	16085	67602	18%	0.0	99.34%	CP043767.1
Leclercia adecarboxylata strain Z96-1 plasmid pZ96-1_1, complete sequence	16085	31193	11%	0.0	99.34%	CP040888.1
Aeromonas salmonicida subsp. pectinolytica 34mel chromosome, complete genome	16085	1.444e+05	23%	0.0	99.34%	CP022426.1
plasmid pFBAOT6 from Aeromonas punctata (Aeromonas caviae) HGB5, complete sequence	16085	53867	16%	0.0	99.34%	CR376602.1
Enterobacter cloacae strain EN3600 plasmid unnamed2, complete sequence	16079	31181	11%	0.0	99.32%	CP035634.1
Aeromonas sp. ASNIH7 chromosome, complete genome	16079	2.107e+05	31%	0.0	99.32%	CP026226.1
Enterobacter kobei strain DSM 13645 chromosome, complete genome	16079	33104	13%	0.0	99.32%	CP017181.1
Citrobacter freundii complex sp. CFNIH3 plasmid pCFR-9161, complete sequence	16078	30338	9%	0.0	99.32%	CP026237.1
Klebsiella pneumoniae strain 1050 plasmid pKp1050-2, complete sequence	16074	35858	11%	0.0	99.31%	CP023418.1
Klebsiella oxytoca strain CAV1374 plasmid pCAV1374-150, complete sequence	16055	17779	7%	0.0	99.28%	CP011633.1
Aeromonas sp. ASNIH4 chromosome, complete genome	16041	75620	21%	0.0	99.25%	CP026217.1
Aeromonas sp. ASNIH5 chromosome, complete genome	15612	1.282e+05	32%	0.0	98.36%	CP026122.1
Klebsiella oxytoca strain AR_0028 plasmid unitig_2_pilon, complete sequence	15394	33438	11%	0.0	97.92%	CP026717.1
Escherichia coli strain MDR_56 plasmid unnamed1, complete sequence	15306	16428	6%	0.0	97.74%	CP019904.1
Klebsiella pneumoniae subsp. ozaenae strain AR_0096 plasmid unnamed2, complete sequence	15300	33344	11%	0.0	97.73%	CP027614.1

Description	Max Score	Total Score	Query Cover	E value	Per. Ident	Accession
Pseudomonas aeruginosa DNA, complete genome, strain: NCGM257	14345	62209	16%	0.0	99.27%	AP014651.1
Serratia marcescens strain BWH-35 plasmid unnamed, complete sequence	13784	20082	7%	0.0	99.16%	CP020508.1
Serratia marcescens strain 95 plasmid unnamed1, complete sequence	13784	20082	7%	0.0	99.16%	CP020506.1
Klebsiella oxytoca strain KONIH2 plasmid pKOR-e3cb, complete sequence	13784	21982	8%	0.0	99.16%	CP026282.1
Raoultella electrica strain DSM 102253 plasmid unnamed1, complete sequence	13701	21899	8%	0.0	99.16%	CP041248.1
Citrobacter freundii complex sp. CFNIH4 plasmid pCFR-4109, complete sequence	13701	21899	8%	0.0	99.16%	CP026234.1
Aminobacter sp. MSH1 chromosome, complete genome	13285	22666	9%	0.0	96.53%	CP026265.1
Aminobacter sp. MSH1 chromosome, complete genome	13285	22666	9%	0.0	96.53%	CP028968.1
Enterobacter hormaechei strain E5 chromosome, complete genome	12855	23407	8%	0.0	99.12%	CP042571.1
Aeromonas hydrophila strain D4 plasmid pAhD4-1, complete sequence	9816	49547	28%	0.0	86.33%	CP013966.1
Aeromonas hydrophila NJ-35, complete genome	9760	57532	32%	0.0	86.22%	CP006870.1
Aeromonas caviae strain WCW1-2 chromosome, complete genome	9334	1.024e+05	17%	0.0	99.96%	CP039832.1
Vibrio parahaemolyticus plasmid pVPS129, complete sequence	9332	40289	14%	0.0	100.00%	KY014464.1
Raoultella ornithinolytica strain WLK218 plasmid pWLK-NDM, complete sequence	9321	18434	6%	0.0	99.94%	CP038280.1
Providencia sp. WCHPHu000369 strain WCHPr000369 plasmid pIMP69_000369, complete sequence	9274	35591	15%	0.0	99.78%	CP031122.1
Klebsiella pneumoniae strain 13294 plasmid p13294-KPC, complete sequence	9140	27928	10%	0.0	97.14%	MF156708.1
Klebsiella quasipneumoniae strain A708 plasmid pA708-1, complete sequence	9023	28275	8%	0.0	99.98%	CP026369.1
Klebsiella aerogenes strain AR_0161 plasmid unnamed, complete sequence	9023	35856	11%	0.0	99.98%	CP028952.1
Klebsiella pneumoniae strain A708 plasmid pA708-IMP, complete sequence	9023	28275	8%	0.0	100.00%	MF344567.1
Klebsiella pneumoniae strain KP1814 plasmid pKP1814-1, complete sequence	9023	34829	11%	0.0	99.98%	KX839207.1
Enterobacter hormaechei strain T5282 plasmid pT5282-Ct2, complete sequence	8883	49728	19%	0.0	99.81%	MF344574.1
Citrobacter freundii strain UMH19 plasmid pUMH19, complete sequence	8776	10816	4%	0.0	98.10%	CP024674.1
Citrobacter freundii strain UMH19 chromosome, complete genome	8776	26799	10%	0.0	98.10%	CP024673.1
Klebsiella pneumoniae strain Kpn-431, plasmid pKPN-431cz, complete sequence	8770	54285	12%	0.0	98.06%	KY020154.1
Escherichia coli strain ECONIH4 plasmid pECO-816c, complete sequence	8610	10648	4%	0.0	97.50%	CP026403.1
Escherichia coli strain ECONIH4 plasmid pKPC-b33e, complete sequence	8610	10648	4%	0.0	97.50%	CP026401.1
Escherichia coli strain ECONIH5 plasmid pKPC-e3ee, complete sequence	8582	10620	4%	0.0	97.37%	CP026205.1
Escherichia coli strain ECONIH5 plasmid pKPC-bca9, complete sequence	8576	17028	7%	0.0	97.36%	CP026204.1
Leclercia adecarboxylata strain E1 plasmid pE1_002, complete sequence	8501	21628	8%	0.0	98.79%	CP042507.1
Citrobacter sp. CF971 plasmid pBM527-4, complete sequence	8495	22975	9%	0.0	98.76%	CP041050.1
Enterobacter cloacae strain 109 chromosome, complete genome	8495	13966	5%	0.0	98.79%	CP020525.1

Description	Max Score	Total Score	Query Cover	E value	Per. Ident	Accession
Leclercia sp. LSNIH3 plasmid pLEC-5e18, complete sequence	8490	21617	8%	0.0	98.76%	CP026390.1
Pseudomonas aeruginosa strain H25883 chromosome, complete genome	8466	14334	6%	0.0	96.86%	CP033686.1
Pseudomonas aeruginosa strain PA83, complete genome	8447	50227	9%	0.0	96.84%	CP017293.1
Shewanella algae strain KC-Na-R1 plasmid pKC-Na-R1, complete sequence	8196	46877	10%	0.0	95.89%	CP033574.1
Klebsiella michiganensis strain FDAARGOS_647 chromosome, complete genome	8161	11881	5%	0.0	95.89%	CP044109.1
Enterobacter hormaechei subsp. steigerwaltii isolate C309 genome assembly, plasmid: pC309-p2	8161	13777	6%	0.0	95.89%	LT991956.1
Citrobacter sp. CFNIH10 chromosome, complete genome	8161	21763	4%	0.0	95.89%	CP026216.1
Klebsiella oxytoca strain AR_0147, complete genome	8161	11948	5%	0.0	95.89%	CP020358.1
Citrobacter freundii plasmid pKHM-1 DNA, complete sequence, strain: KHM 243	8161	49393	22%	0.0	95.89%	AP014939.1
Pseudomonas aeruginosa strain RJ248 class 1 integron ISCR1 PER-1, partial sequence	8021	22946	6%	0.0	99.93%	KU133340.1
Salmonella enterica subsp. enterica serovar Dublin plasmid pMAK3 DNA, complete genome, strain: L-2156	7993	24906	6%	0.0	99.88%	AB366442.1
Escherichia coli strain A1_180 plasmid unnamed1, complete sequence	7986	48423	9%	0.0	99.88%	CP040382.1
Enterobacter cloacae strain 174 plasmid unnamed1, complete sequence	7986	47023	8%	0.0	99.88%	CP020529.1
Enterobacter hormaechei strain S13 plasmid pSHV12-1301491, complete sequence	7986	55384	9%	0.0	99.88%	CP031568.1
Salmonella enterica subsp. enterica serovar Newport strain 0307-213, complete genome	7986	64346	15%	0.0	99.88%	CP012599.1
Enterobacter hormaechei subsp. steigerwaltii strain 34977 plasmid p34977-263.138kb, complete sequence	7986	49996	9%	0.0	99.88%	CP012170.1
Serratia marcescens SM39 plasmid pSMC1 DNA, complete genome	7986	32690	8%	0.0	99.93%	AP013064.1
Klebsiella pneumoniae plasmid pGDKA1 class 1 sul1-type integron intI1 (intI1) gene, partial cds, and dihydrofolate reductase (dfrA27), AADA2-aminoglycoside-(3")(9)-adenylyltransferase (aadA2), quaternary ammonium compound resistance protein (qacEdelta1), Sul1 (sul1), putative recombinase (ISCR1), QnrA1 (qnrA1), and Sul1 (sul1) genes, complete cds; aminoglycoside 3'-phosphotransferase type 1 (aph(3')-I) gene, complete cds; transposase pseudogene, complete sequence; insertion sequence IS26 TnpA (TnpA) gene, complete cds; ImpB/MucB/SamB family protein pseudogene, complete sequence; ParB (parB) gene, complete cds; and ParA (parA) gene, partial cds	7986	22872	6%	0.0	99.91%	EU722351.3
Enterobacter hormaechei subsp. hoffmannii strain AR_0365 plasmid unnamed1, complete sequence	7984	37183	9%	0.0	99.93%	CP027144.1
Raoultella ornithinolytica strain 23141 plasmid p23141-3, complete sequence	7984	21502	8%	0.0	99.93%	MF788071.1
Phytobacter ursingii strain CAV1151 plasmid pCAV1151-296, complete sequence	7984	36821	10%	0.0	99.93%	CP011601.1
Escherichia coli plasmid pSa, partial sequence	7984	25961	6%	0.0	99.95%	L06822.4
Enterobacter hormaechei strain C15 plasmid pC15_001, complete sequence	7982	46049	8%	0.0	99.95%	CP042489.1
Enterobacter cloacae strain EC62 plasmid pIMP-4-EC62, complete sequence	7982	48178	8%	0.0	99.93%	MH829594.1

Description	Max Score	Total Score	Query Cover	E value	Per. Ident	Accession
Enterobacter hormaechei strain WCHEH020038 plasmid pCTXM9_020038, complete sequence	7982	57457	10%	0.0	99.98%	CP031724.1
Enterobacter cloacae complex bacterium isolate C45 genome assembly, plasmid: pC45-VIM4	7982	57976	14%	0.0	99.98%	LT991958.1
Klebsiella pneumoniae strain 721005, complete genome	7982	17779	6%	0.0	99.95%	CP022997.1
Citrobacter freundii strain Cf52 plasmid pCf52, complete sequence	7982	82660	17%	0.0	99.93%	KY887592.1
Salmonella enterica subsp. enterica serovar Infantis strain SRC46 Salmonella Genomic Island 1 variant SGI1-D, complete sequence	7982	25186	6%	0.0	99.93%	KU854986.1
Salmonella enterica subsp. enterica serovar Bovismorbificans strain HP507391 plasmid IncHI2 In60-like integron, complete sequence	7982	16187	5%	0.0	99.98%	JX026665.1
Klebsiella pneumoniae strain Kp1206 plasmid p1206 class 1 integron, partial sequence	7982	16370	5%	0.0	99.93%	EU622038.1
Aeromonas hydrophila plasmid pRA3, complete sequence	7982	24501	6%	0.0	99.95%	DQ401103.1
Enterobacter hormaechei subsp. steigerwaltii strain ME-1 plasmid pME-1a, complete sequence	7980	45666	8%	0.0	99.98%	CP041734.1
Salmonella enterica subsp. enterica serovar 4,[5],12:i:- strain 77 plasmid, partial sequence	7980	54842	10%	0.0	99.86%	MK191844.1
Vibrio alginolyticus strain Vb1394 plasmid pC1394, complete sequence	7980	48856	13%	0.0	99.98%	MH457126.1
Escherichia coli strain 511 genome assembly, plasmid: RCS54_p	7980	18321	6%	0.0	99.98%	LT985263.1
Escherichia coli strain DSM 103246, complete genome	7980	31290	10%	0.0	99.98%	CP019944.1
Enterobacter hormaechei strain 917144 class 1 integron dihydrofolate reductase (dfr16), aminoglycoside-(3")(9)-adenylyltransferase (aadA2), quaternary ammonium compound-resistance protein (qacEdelta1), dihydropteroate synthase (sul1), putative recombinase, QnrA1 (qnrA1), and AmpR (ampR) genes, complete cds	7980	17101	5%	0.0	99.98%	KC414000.1
Enterobacter cloacae strain YMC01/10/R768 class 1 integron, partial sequence	7980	22032	5%	0.0	99.98%	HQ184955.1
Klebsiella oxytoca plasmid pK9AT DNA, class I integron InK9AT, partial sequence	7980	17834	6%	0.0	99.98%	AB469045.1
Enterobacter cloacae plasmid pEBQ-1 class 1 integron IntI1 (intI1) gene, partial cds, Dfr15 (dfr15), AadA2 (aadA2), QacEdeltal (qacEdeltal), Sul1 (sul1), QnrA1 (qnrA1), and AmpR (ampR) genes, complete cds, and unknown gene	7980	16498	5%	0.0	99.98%	DQ989302.1
Escherichia coli plasmid pHSH1 encoded class 1 integron In36, complete sequence	7980	24887	6%	0.0	99.98%	AY259085.1
Salmonella enterica subsp. enterica serovar Braenderup strain 76 plasmid, partial sequence	7978	55760	10%	0.0	99.91%	MK191835.1
Klebsiella pneumoniae plasmid pKP1334/05-1, partial sequence	7978	22307	5%	0.0	99.91%	FJ943244.1
Salmonella enteritidis plasmid pSAL-1 integron, ampC and ampR genes (beta-lactamase DHA-1), aadA2 gene, ORF 341, strain KF92	7978	22466	5%	0.0	99.91%	AJ237702.1
Salmonella enterica subsp. enterica serovar Keurmassar partial qnr-containing complex sul1-type integron	7978	32107	6%	0.0	99.86%	AM234698.1
Enterobacter cloacae strain MRSN17626 plasmid pMRVIM0813, complete sequence	7976	55115	11%	0.0	99.95%	KP975077.1
Klebsiella pneumoniae partial pKp760 plasmid	7976	22873	6%	0.0	99.95%	AJ971341.1

BLAST ® » **blastn suite** » results for RID-VGJ6P1NA014

Job Title gb|EF495198.1| ...
RID VGJ6P1NA014 Search expires on 10-30 22:12 pm
Program BLASTN
Database nt
Query ID EF495198.1
Description Aeromonas bestiarum plasmid pAb5S9, complete sequence ...
Molecule type nucleic acid
Query Length 24716

Descriptions

Description	Max Score	Total Score	Query Cover	E value	Per. Ident	Accession
Aeromonas bestiarum plasmid pAb5S9, complete sequence	45642	47569	100%	0.0	100.00%	EF495198.1
TPA: Aeromonas bestiarum strain 5S9 plasmid pAb5S9, complete sequence	41918	47570	100%	0.0	100.00%	BK008853.1
Aeromonas salmonicida subsp. salmonicida strain 2009-144K3 plasmid pAB5S9b, complete sequence	19241	43050	90%	0.0	99.93%	KJ909292.1
Escherichia coli strain YSP8-1 plasmid pYSP8-1, complete sequence	9884	19238	22%	0.0	99.83%	CP037911.1
Uncultured bacterium IN-12 genomic sequence	9177	18815	41%	0.0	99.80%	KU736877.1
Proteus mirabilis integrative and conjugative element ICEPmiChn4, complete sequence; and peptide chain release factor 3 (prfC) gene, complete cds	8892	16160	31%	0.0	99.96%	KY437728.1
Shewanella upenei strain 110003 ICESupCHN110003 mobile element, complete sequence	8887	16177	31%	0.0	99.94%	MG014393.1
Citrobacter sp. SNU WT2 plasmid unnamed1, complete sequence	8881	16177	31%	0.0	99.92%	CP038468.1
Vibrio cholerae strain E4 chromosome 1, complete sequence	8881	16171	31%	0.0	99.92%	CP033515.1
Vibrio cholerae strain IDH_4268 SXT element genomic sequence	8881	16171	31%	0.0	99.92%	MK165650.1
Vibrio cholerae O1 biovar El Tor ICE-element genomic island sequence	8881	16621	31%	0.0	99.92%	MG950412.1
Escherichia coli strain 11011 plasmid p11011-fosA, complete sequence	8881	16177	31%	0.0	99.92%	MG764548.1
Vibrio cholerae O1 biovar El Tor strain HC1037 chromosome I, complete sequence	8881	16171	31%	0.0	99.92%	CP026647.1
Proteus mirabilis strain AR_0159, complete genome	8881	16177	31%	0.0	99.92%	CP021550.1
Vibrio cholerae O1 biovar El Tor str. Ogawa RND6878 ICE element, complete sequence	8881	16171	31%	0.0	99.92%	KY382507.1
Proteus mirabilis strain MD20140905 complete sequence	8881	16171	31%	0.0	99.92%	KX243412.1
Proteus mirabilis strain MD20140904 complete sequence	8881	16171	31%	0.0	99.92%	KX243411.1
Vibrio sp. 85(2016) genomic sequence	8881	17892	31%	0.0	99.92%	KU306395.1
Vibrio cholerae O1 str. KW3 chromosome I, complete sequence	8881	16171	31%	0.0	99.92%	CP006947.1
Vibrio cholerae O1 biovar El Tor strain FJ147 chromosome I, complete sequence	8881	16171	31%	0.0	99.92%	CP009042.1
Vibrio cholerae VC833 ICE element genomic sequence	8881	16171	31%	0.0	99.92%	KC886258.1
Vibrio cholerae strain VC504 ICE element genomic sequence	8881	16171	31%	0.0	99.92%	KC886257.1

Description	Max Score	Total Score	Query Cover	E value	Per. Ident	Accession
Vibrio cholerae strain 2012EL-2176 chromosome 1, complete sequence	8881	32343	31%	0.0	99.92%	CP007634.1
Vibrio cholerae strain VC1786ICE genomic sequence	8881	16171	31%	0.0	99.92%	JN648379.1
Vibrio cholerae O1 str. 2010EL-1786 chromosome 1, complete sequence	8881	16171	31%	0.0	99.92%	CP003069.1
Vibrio cholerae O1 biovar El Tor SXT conjugative element gene cluster, complete sequence, strain: KN14	8881	16171	31%	0.0	99.92%	AB535680.1
Vibrio cholerae Ban5 integrating conjugative element ICEVchban5, complete sequence	8881	25029	31%	0.0	99.92%	GQ463140.1
Vibrio cholerae Ind5 integrating conjugative element ICEVchind5, complete sequence	8876	16166	31%	0.0	99.90%	GQ463142.1
Vibrio cholerae Ind4 integrating conjugative element ICEVchind4, complete sequence	8876	16171	31%	0.0	99.90%	GQ463141.1
Actinobacillus pleuropneumoniae strain MIDG3553 YhaH (yhaH) gene, complete cds; ICEApl2 mobile element, complete sequence; and PrfC (prfC) gene, complete cds	8874	16162	31%	0.0	99.90%	MF187965.1
Providencia alcalifaciens Ban1 integrating conjugative element ICEPalban1, complete sequence	8874	16170	31%	0.0	99.90%	GQ463139.1
Vibrio cholerae DNA, integrating antibiotic resistance gene element	8870	17881	31%	0.0	99.88%	AB114188.1
Salmonella enterica subsp. enterica serovar 4,[5],12:i:- strain 77 plasmid, partial sequence	8868	18641	31%	0.0	99.96%	MK191844.1
Salmonella enterica subsp. enterica serovar Braenderup strain 76 plasmid, partial sequence	8868	18641	31%	0.0	99.96%	MK191835.1
Aeromonas salmonicida strain S44 plasmid pS44-1, complete sequence	8868	15473	26%	0.0	99.96%	CP022176.1
Proteus mirabilis integrative and conjugative element ICEPmiChn3, complete sequence; and peptide chain release factor 3 (prfC) gene, complete cds	8868	15375	24%	0.0	99.96%	KY437727.1
Vibrio parahaemolyticus strain VPS92 plasmid pVPS92-VEB, complete sequence	8868	17890	31%	0.0	99.88%	KU356480.1
Proteus mirabilis integrating conjugative element ICEPmiChn-BCP11 sequence	8865	20257	31%	0.0	99.83%	MG773277.1
Proteus mirabilis strain PmPHI integrative and conjugative element ICEPmiFra1 mobile element, complete sequence; and peptide chain release factor 3 (prfC) gene, complete cds	8865	16160	31%	0.0	99.85%	MF490434.1
Escherichia coli strain 2016C-3878 plasmid pMCR1-PA, complete sequence	8863	26548	31%	0.0	99.94%	CP029748.1
Shewanella putrefaciens strain NCTC12093 genome assembly, chromosome: 1	8857	23690	31%	0.0	99.92%	LR134303.1
Edwardsiella piscicida strain ETW41 plasmid pETW41 sequence	8857	15353	24%	0.0	99.92%	CP019441.1
Acinetobacter pittii strain AB17H194 plasmid p13C018-1, complete sequence	8855	21642	31%	0.0	99.83%	CP040912.1
Proteus mirabilis strain PmSC1111 chromosome, complete genome	8853	37991	31%	0.0	99.79%	CP034090.1
Escherichia coli strain ECCWS199 plasmid pTB222, complete sequence	8852	15353	24%	0.0	99.90%	CP032239.1
Proteus mirabilis integrative and conjugative element ICEPmiChn2, complete sequence; and peptide chain release factor 3 (prfC) gene, complete cds	8852	15340	24%	0.0	99.90%	KY437726.1
Klebsiella pneumoniae strain AR_0152 plasmid tig00000195, complete sequence	8852	15335	24%	0.0	99.90%	CP021946.1
Escherichia coli strain SCEC020023 plasmid pOXA10_020023, complete sequence	8846	15324	24%	0.0	99.88%	CP025944.4

Description	Max Score	Total Score	Query Cover	E value	Per. Ident	Accession
Enterobacter hormaechei strain SCEH020042 plasmid pQnrB4_020042, complete sequence	8846	18614	31%	0.0	99.88%	CP028537.1
Vibrio cholerae O1 biovar El Tor str. Inaba RND18826 ICE element, complete sequence	8844	16098	31%	0.0	99.92%	KY382506.1
Escherichia coli strain cq9 plasmid unnamed3, complete sequence	8841	24184	31%	0.0	99.85%	CP031549.1
Rheinheimera sp. D18 chromosome, complete genome	8828	33906	31%	0.0	99.81%	CP037745.1
Edwardsiella piscicida strain MS-18-199 plasmid pEP-MS-18-199, complete sequence	8791	16064	31%	0.0	99.59%	CP035669.1
Acinetobacter baumanii ISCR2 element, isolate ABC23 from Argentina	8730	14437	22%	0.0	99.89%	FN293050.1
Escherichia coli strain NCYU-21-79 plasmid pNCYU-21-79-1, complete sequence	8728	15134	24%	0.0	99.42%	CP042646.1
Vibrio sp. 63(2016) genomic sequence	8663	15172	30%	0.0	99.91%	KU306394.1
Escherichia coli O157 strain AR-0429 plasmid pAR-0429-1, complete sequence	8468	15368	31%	0.0	99.96%	CP044142.1
Proteus mirabilis strain CRPM10 chromosome, complete genome	8468	10004	19%	0.0	99.93%	CP043332.1
Aeromonas hydrophila strain 23-C-23 plasmid unnamed, complete sequence	8468	15368	31%	0.0	99.96%	CP038466.1
Aeromonas hydrophila strain WCX23 plasmid unnamed, complete sequence	8468	15368	31%	0.0	99.96%	CP038464.1
Aeromonas hydrophila strain WCX23 plasmid pWCX23_1, complete sequence	8468	15368	31%	0.0	99.93%	CP028419.1
Escherichia coli strain AMSCJX02 plasmid pAMSC1, complete sequence	8468	15357	31%	0.0	99.93%	CP031106.1
Salmonella enterica strain SA20025921 plasmid pSA20025921.1, complete sequence	8468	15368	31%	0.0	99.96%	CP030215.1
Salmonella enterica subsp. enterica serovar London strain Sa128 plasmid pSa128, complete sequence	8468	15366	31%	0.0	99.96%	MG870194.1
Vibrio alginolyticus strain Vb1796 plasmid pVb1796, complete sequence	8468	15368	31%	0.0	99.96%	MH113855.1
Salmonella sp. strain Sa63 plasmid pSa63-CIP, complete sequence	8468	15366	31%	0.0	99.93%	MG874043.1
Aeromonas salmonicida subsp. salmonicida strain 2004-05MF26 plasmid pSN254b, complete sequence	8468	15368	31%	0.0	99.96%	KJ909290.1
Escherichia coli strain PG010208 plasmid pPG010208, complete sequence	8468	15368	31%	0.0	99.96%	HQ023861.1
Escherichia coli strain ECZP248 plasmid pTB402, complete sequence	8462	15353	31%	0.0	99.91%	CP034786.1
Escherichia coli strain 14EC047 plasmid p14EC047b, complete sequence	8462	9999	19%	0.0	99.91%	CP024157.1
Escherichia coli GSH8M-2 plasmid pGSH8M-2-1 DNA, complete genome	8456	9993	19%	0.0	99.89%	AP019676.1
Klebsiella pneumoniae strain 130411-38618 plasmid p130411-38618_1, complete sequence	8456	15350	31%	0.0	99.89%	MK649826.1
Salmonella enterica subsp. enterica strain AR-0401 plasmid pAR-0401-1, complete sequence	8456	15357	31%	0.0	99.89%	CP044189.1
Escherichia coli O16:H48 strain PG20180173 plasmid pPG20180173.1-IncAC2, complete sequence	8456	15357	31%	0.0	99.89%	CP043192.1
Escherichia coli O16:H48 strain PG20180175 plasmid pPG20180175.1-IncAC2, complete sequence	8456	15357	31%	0.0	99.89%	CP043190.1
Salmonella enterica subsp. enterica serovar Heidelberg strain SL-312 plasmid pET8.1-IncAC2, complete sequence	8456	15357	31%	0.0	99.89%	CP043215.1

Description	Max Score	Total Score	Query Cover	E value	Per. Ident	Accession
Escherichia coli strain LD91-1 plasmid pLD91-1-146kb, complete sequence	8456	9993	19%	0.0	99.89%	CP042586.1
Klebsiella pneumoniae strain Kp202 plasmid pKp202_1, complete sequence	8456	15357	31%	0.0	99.91%	CP041083.1
Escherichia coli strain L725 plasmid punnamed3, complete sequence	8456	15355	31%	0.0	99.91%	CP036206.1
Escherichia coli strain L73 plasmid pL73-2, complete sequence	8456	15311	31%	0.0	99.91%	CP033379.1
Klebsiella pneumoniae strain MSB1_8A-sc-2280397 plasmid unnamed2, complete sequence	8456	17521	31%	0.0	99.91%	CP031802.1
Proteus mirabilis strain CA150272 proteus genomic island 2 PGI2-PmCA72, complete sequence	8456	9995	19%	0.0	99.89%	MH990678.1
Salmonella enterica subsp. enterica serovar Newport str. USDA-ARS-USMARC-1925 plasmid pSNE1-1925, complete sequence	8456	16377	30%	0.0	99.89%	CP025233.1
Citrobacter freundii isolate Citrobacter freundii str. U2785 genome assembly, plasmid: 2	8456	25351	31%	0.0	99.91%	LS992184.1
Escherichia coli strain L65 plasmid pL65-2, complete sequence	8456	9993	19%	0.0	99.89%	CP034739.1
Escherichia coli strain WCHEC005237 plasmid pQnrS1_005237, complete sequence	8456	9993	19%	0.0	99.89%	CP026578.2
Proteus mirabilis strain CCUG 70746 plasmid pPmi70746_1, complete sequence	8456	15357	31%	0.0	99.89%	CP023274.1
Klebsiella pneumoniae strain 13190 plasmid p13190-tetA, complete sequence	8456	12670	25%	0.0	99.91%	MG764549.1
Salmonella enterica subsp. enterica serovar Corvallis strain 12-01738 plasmid pSE12-01738-2, complete sequence	8456	15357	31%	0.0	99.89%	CP027679.1
Escherichia coli strain Esco-36073cz plasmid pEsco-36073cz, complete sequence	8456	15357	31%	0.0	99.91%	MG252895.1
Escherichia coli strain AMA566 plasmid pAMA566, complete sequence	8456	15357	31%	0.0	99.91%	MG450360.1
Escherichia coli strain 92944 plasmid p92944-TEM, complete sequence	8456	18108	30%	0.0	99.89%	MG860488.1
Salmonella enterica subsp. enterica serovar Concord strain CFSAN018747 plasmid pGMI14-002_1, complete sequence	8456	28409	31%	0.0	99.91%	CP028197.1
Escherichia coli plasmid pBJ114T-190, complete sequence	8456	15351	31%	0.0	99.89%	MF679147.1
Escherichia coli plasmid pBJ114-141, complete sequence	8456	15278	31%	0.0	99.89%	MF679146.1
Escherichia coli strain 14EC020 plasmid p14EC020b, complete sequence	8456	14834	30%	0.0	99.91%	CP024140.1
Klebsiella pneumoniae strain 825795-1 plasmid unnamed2, complete sequence	8456	15421	31%	0.0	99.91%	CP017987.1
Proteus mirabilis strain TJ3335 complete sequence	8456	15344	31%	0.0	99.91%	KX243416.1
Klebsiella pneumoniae strain Kp_Goe_152021 plasmid pKp_Goe_021-2, complete sequence	8456	15421	31%	0.0	99.91%	CP018716.1
Klebsiella pneumoniae strain Kp_Goe_827026 plasmid pKp_Goe_026-2, complete sequence	8456	15421	31%	0.0	99.91%	CP018710.1

Graphic Summary

BLAST® » blastn suite » results for RID-VGJMYA07014

Job Title	JN315884:Aeromonas sobria plasmid pAQ2-1,... ...
RID	VGJMYA07014 Search expires on 10-30 22:20 pm
Program	BLASTN
Database	nt
Query ID	JN315884.1
Description	Aeromonas sobria plasmid pAQ2-1, complete sequence ...
Molecule type	nucleic acid
Query Length	6900

Descriptions

Description	Max Score	Total Score	Query Cover	E value	Per. Ident	Accession
Aeromonas sobria plasmid pAQ2-1, complete sequence	12743	12743	100%	0.0	100.00%	JN315884.1
Aeromonas hydrophila plasmid pAQ2-2, complete sequence	12429	12429	100%	0.0	99.17%	JN315885.1
Uncultured prokaryote from Rat gut metagenome metamobilome, plasmid pRGRH0380	4837	7092	58%	0.0	97.47%	LN853036.1
Aeromonas sp. ASNIH2 plasmid pAER-0ed9, complete sequence	3696	7736	65%	0.0	96.01%	CP026411.1
Aeromonas caviae isolate AB5 plasmid pAB5, complete sequence	2525	2525	19%	0.0	99.93%	KU644674.1
Enterobacter kobei strain WCHEK045523 plasmid pQnrS2_045523, complete sequence	2521	2521	19%	0.0	99.93%	CP032896.1
Citrobacter freundii strain AA593 plasmid pIBAC_Incx3_A/C, complete sequence	2521	2521	19%	0.0	99.93%	MH594478.1
Aeromonas taiwanensis strain L1713 plasmid p1713-KPC, complete sequence	2521	2521	19%	0.0	99.93%	MH624132.1
Aeromonas taiwanensis strain L198 plasmid p198-KPC, complete sequence	2521	2521	19%	0.0	99.93%	MH624131.1
Aeromonas taiwanensis strain L186 plasmid p186-KPC, complete sequence	2521	2521	19%	0.0	99.93%	MH624130.1
Aeromonas sp. ASNIH2 plasmid pAER-e58e, complete sequence	2521	2521	19%	0.0	99.93%	CP026410.1
Aeromonas caviae isolate HP2 plasmid pHP2, complete sequence	2521	2521	19%	0.0	99.93%	KU644677.1

Description	Max Score	Total Score	Query Cover	E value	Per. Ident	Accession
Aeromonas allosaccharophila isolate HP5 plasmid pHP5, complete sequence	2521	2521	19%	0.0	99.93%	KU644676.1
Aeromonas caviae isolate HP1 plasmid pHP1, complete sequence	2521	2521	19%	0.0	99.93%	KU644673.1
Aeromonas caviae isolate HP18 plasmid pPH18, complete sequence	2521	2521	19%	0.0	99.93%	KU644672.1
Klebsiella pneumoniae strain I212 plasmid pKPSH212, complete sequence	2521	2576	19%	0.0	99.93%	KT896501.1
Aeromonas sobria strain ASCH21 plasmid pASCH21, complete sequence	2521	2521	19%	0.0	99.93%	KT315928.1
Aeromonas hydrophila strain AH227 plasmid pAH227, complete sequence	2521	2521	19%	0.0	99.93%	KT315926.1
Uncultured bacterium plasmid pKAZ5, complete sequence	2521	2521	19%	0.0	99.93%	KR827394.1
Aeromonas hydrophila strain IB101 plasmid AHIB101-pBF7.8, complete sequence	2521	2521	19%	0.0	99.93%	KM245123.1
Aeromonas punctata plasmid p37 quinolone resistance protein S2 (qnrS2) gene, complete cds; and disrupted neutral zinc metallopeptidase (mpR) gene, partial sequence	2521	2521	19%	0.0	99.93%	EU439940.1
Salmonella enterica subsp. enterica serovar Typhimurium var. 5- strain 63 plasmid, partial sequence	2316	2316	18%	0.0	100.00%	MK191840.1
Klebsiella pneumoniae strain I231 plasmid pKPSH231, complete sequence	2316	2316	18%	0.0	100.00%	KT896503.1
Klebsiella pneumoniae strain I70 plasmid pKPSH70, complete sequence	2316	2316	18%	0.0	100.00%	KT896500.1
Klebsiella pneumoniae strain I169 plasmid pKPSH169, complete sequence	2316	2316	18%	0.0	100.00%	KT896499.1
uncultured bacterium clone AA-101 plasmid pFECG, complete sequence	2270	2506	18%	0.0	99.92%	MF554638.1
Aeromonas caviae isolate HP16 plasmid pHP16, complete sequence	2224	2224	17%	0.0	100.00%	KU644675.1

Description	Max Score	Total Score	Query Cover	E value	Per. Ident	Accession
Salmonella enterica subsp. enterica serovar Typhimurium strain SH16G4525 plasmid pSH16G4525, complete sequence	2196	2196	17%	0.0	100.00%	MH522424.1
Salmonella enterica subsp. enterica serovar Typhimurium strain SH16G4498 plasmid pSH16G4498, complete sequence	2196	2196	17%	0.0	100.00%	MH522423.1
Salmonella enterica subsp. enterica serovar Typhimurium strain SH16G2457 plasmid pSH16G2457, complete sequence	2196	2196	17%	0.0	100.00%	MH522421.1
Salmonella enterica subsp. enterica serovar Typhimurium strain SH16G1508 plasmid pSH16G1508, complete sequence	2196	2196	17%	0.0	100.00%	MH522420.1
Escherichia fergusonii strain EFCF056 plasmid pEF05, complete sequence	2196	2196	17%	0.0	100.00%	CP040810.1
Escherichia fergusonii strain EFCF056 plasmid pEF04, complete sequence	2196	2196	17%	0.0	100.00%	CP040809.1
Salmonella enterica subsp. enterica serovar California 2,4-dienoyl-CoA reductase [NADPH] (fadH), Putrescine aminotransferase (yjiG), and Hypothetical protein genes, complete cds; multidrug resistance gene cluster, complete sequence; and Methyl-accepting chemotaxis protein II gene, complete cds	2196	2196	17%	0.0	100.00%	MH079550.1
Salmonella enterica subsp. enterica strain CFSA231 chromosome, complete genome	2196	2196	17%	0.0	100.00%	CP033350.2
Salmonella enterica subsp. enterica serovar California strain CD-SL01 chromosome, complete genome	2196	2196	17%	0.0	100.00%	CP028900.1
Escherichia coli strain FS11Y5C plasmid pFS11Y5CT, complete sequence	2196	2196	17%	0.0	100.00%	MG014721.1
Salmonella enterica subsp. enterica serovar Derby strain Sa64 plasmid pSa64T-188, complete sequence	2196	2196	17%	0.0	100.00%	CP034251.1

Description	Max Score	Total Score	Query Cover	E value	Per. Ident	Accession
Salmonella enterica subsp. enterica serovar Derby strain Sa64 chromosome, complete genome	2196	2196	17%	0.0	100.00%	CP034250.1
Escherichia coli strain BE2-5 plasmid p2_BE2-5, complete sequence	2196	2196	17%	0.0	100.00%	CP032988.1
Escherichia coli strain 974 plasmid p974-IncF, complete sequence	2196	2196	17%	0.0	100.00%	MH580300.1
Escherichia coli strain RJ749 plasmid pRJ749, complete sequence	2196	2196	17%	0.0	100.00%	MH491004.1
Escherichia coli strain 14EC033 plasmid p14EC033f, complete sequence	2196	2196	17%	0.0	100.00%	CP024153.1
Aeromonas hydrophila strain AH6 plasmid pAH6, complete sequence	2196	2196	17%	0.0	100.00%	KT315927.1
Escherichia coli GSH8M-2 plasmid pGSH8M-2-3 DNA, complete genome	2189	2189	17%	0.0	100.00%	AP019678.1
Escherichia coli strain D72 plasmid pD72-IncX1, complete sequence	2189	2189	17%	0.0	100.00%	CP035315.1
Escherichia coli strain HS13-1 plasmid pHS13-1-IncHI2, complete sequence	2189	2189	17%	0.0	100.00%	CP026492.1
Escherichia coli strain D9 plasmid C, complete genome	2189	2189	17%	0.0	100.00%	CP010155.1
Escherichia coli strain D4, complete genome	2189	2189	17%	0.0	100.00%	CP010143.1
Aeromonas sp. C3 plasmid pAC3, complete sequence	2132	2530	19%	0.0	99.91%	KM204147.1
Aeromonas media plasmid p42 quinolone resistance protein S2 (qnrS2) gene, complete cds; and disrupted neutral zinc metallopeptidase (mpR) gene, partial sequence	2128	2509	19%	0.0	99.91%	EU439941.1
Escherichia coli strain INSRA19829 plasmid pUR19829-KPC21, complete sequence	2108	2323	18%	0.0	99.91%	MH133192.1
Aeromonas hydrophila strain D4 plasmid pAhD4-1, complete sequence	2023	2197	15%	0.0	100.00%	CP013966.1
Aeromonas rivipollensis plasmid pP2G1, complete sequence	1995	2545	19%	0.0	100.00%	HE616910.2

Description	Max Score	Total Score	Query Cover	E value	Per. Ident	Accession
Pseudoalteromonas marina strain E8 TetR family transcriptional regulator (tetR) gene, partial cds; integrase, hypothetical protein, integrase, quinolone resistance protein QnrS2 (qnrS2), Tn7 transposition protein TnsC (tnsC), Tn7 transposition protein TnsB (tnsB), and Tn7 transposition protein TnsA (tnsA) genes, complete cds; and glutamine-fructose-6-phosphate transaminase (isomerizing) (glmS) gene, partial cds	1975	1975	15%	0.0	99.81%	KX756556.1
Pseudoalteromonas marina strain E8 TetR family transcriptional regulator (tetR) gene, partial cds; and integrase, hypothetical protein, integrase, quinolone resistance protein QnrS2 (qnrS2), aminoglycoside nucleotidyltransferase AadA13 (aadA13), trimethoprim-resistant dihydrofolate reductase DfrA6 (dfrA6), and integrase genes, complete cds	1975	1975	15%	0.0	99.81%	KX756555.1
Shewanella colwelliana strain S14 plasmid pS14-1 TetR family transcriptional regulator (tetR), integrase, hypothetical protein, integrase, quinolone resistance protein QnrS2 (qnrS2), and ATPase genes, complete cds	1975	1975	15%	0.0	99.81%	KX756557.1
uncultured bacterium clone AA-102 plasmid pFECR, complete sequence	1973	1973	15%	0.0	99.91%	MF554639.1
Aeromonas hydrophila plasmid pAHH04, complete sequence	1973	2526	19%	0.0	99.91%	JN315883.1
Nitrincola sp. KXZD1103 plasmid unnamed1, complete sequence	1965	1965	15%	0.0	99.81%	CP044223.1

Description	Max Score	Total Score	Query Cover	E value	Per. Ident	Accession
Pseudoalteromonas carrageenovora strain S16 DNA polymerase V (umuD) gene, partial cds; hypothetical protein, endonuclease I (endA), quinolone resistance protein QnrS2 (qnrS2), endonuclease III, electron transport complex subunit RsxE (rsxE), electron transport complex subunit RsxG (rsxG), and electron transport complex subunit RsxD (rsxD) genes, complete cds; and electron transport complex subunit RsxC (rsxC) gene, partial cds	1965	1965	15%	0.0	99.63%	KX756558.1
Aeromonas sp. ASNIH2 plasmid pAER-c633, complete sequence	1940	4117	46%	0.0	88.84%	CP026409.1
Klebsiella pneumoniae strain I213 plasmid pKPSH213.55, complete sequence	1849	2098	16%	0.0	100.00%	KT896502.1
Aeromonas hydrophila strain AO1 plasmid pBRST7.6, complete sequence	1757	2306	18%	0.0	99.79%	EU925817.1
Uncultured bacterium plasmid pGNB2, complete sequence	1659	2210	17%	0.0	99.89%	DQ460733.1
Aeromonas caviae QnrS2 (qnrS2) gene, complete cds	1214	1214	9%	0.0	100.00%	KU644708.1
Salmonella enterica subsp. enterica serovar Anatum pMG308 qnrS gene for quinolone resistance pentapeptide repeat protein QnrS2, complete CDS	1214	1214	9%	0.0	100.00%	NG_050544.1
Salmonella enterica subsp. enterica serovar Anatum plasmid pMG308 QnrS2 (qnrS2) gene, complete cds	1214	1214	9%	0.0	100.00%	DQ485530.1
Aeromonas hydrophila SNUFPC-A5 pAQ2-2 qnrS gene for quinolone resistance pentapeptide repeat protein QnrS6, complete CDS	1208	1208	9%	0.0	99.85%	NG_050547.1
Aeromonas hydrophila strain SNUFPC-A5 plasmid pAQ2-2 quinolone resistance protein gene, complete cds	1208	1208	9%	0.0	99.85%	HQ631376.1
Escherichia coli isolate C1076 quinolone resistance protein (qnrS2) gene, partial cds	1120	1120	8%	0.0	100.00%	KP773325.1

Description	Max Score	Total Score	Query Cover	E value	Per. Ident	Accession
Escherichia coli isolate C1074 quinolone resistance protein (qnrS2) gene, partial cds	1114	1114	8%	0.0	100.00%	KP773324.1
Escherichia coli isolate C705 quinolone resistance protein (qnrS2) gene, partial cds	1114	1114	8%	0.0	100.00%	JF773347.1
Escherichia coli isolate C770 quinolone resistance protein (qnrS2) gene, partial cds	1103	1103	8%	0.0	100.00%	JF773350.1
Escherichia coli isolate C600 quinolone resistance protein (qnrS2) gene, partial cds	1103	1103	8%	0.0	100.00%	JF773346.1
Escherichia coli isolate C598 quinolone resistance protein (qnrS2) gene, partial cds	1103	1103	8%	0.0	100.00%	JF773345.1
Escherichia coli isolate C726 quinolone resistance protein (qnrS2) gene, partial cds	1098	1098	8%	0.0	100.00%	JF773348.1
Escherichia coli isolate C1213 quinolone resistance protein (qnrS2) gene, partial cds	1092	1092	8%	0.0	100.00%	KP773336.1
Escherichia coli isolate C1080 quinolone resistance protein (qnrS2) gene, partial cds	1086	1086	8%	0.0	100.00%	KP773328.1
Escherichia coli isolate C1236 quinolone resistance protein (qnrS2) gene, partial cds	1085	1085	8%	0.0	100.00%	KP773337.1
Escherichia coli isolate C1081 quinolone resistance protein (qnrS2) gene, partial cds	1081	1081	8%	0.0	100.00%	KP773329.1
Escherichia coli isolate C1066 quinolone resistance protein (qnrS2) gene, partial cds	1081	1081	8%	0.0	100.00%	KP773323.1
Escherichia coli isolate C1062 quinolone resistance protein (qnrS2) gene, partial cds	1075	1075	8%	0.0	100.00%	KP773322.1
Escherichia coli isolate C197 quinolone resistance protein (qnrS2) gene, partial cds	1070	1070	8%	0.0	100.00%	JF773343.1
Escherichia coli isolate C387 quinolone resistance protein (qnrS2) gene, partial cds	1064	1064	8%	0.0	100.00%	JF773344.1
Escherichia coli isolate C1100 quinolone resistance protein (qnrS2) gene, partial cds	1048	1048	8%	0.0	100.00%	KP773330.1

Description	Max Score	Total Score	Query Cover	E value	Per. Ident	Accession
Escherichia coli isolate C943 quinolone resistance protein (qnrS2) gene, partial cds	1048	1048	8%	0.0	99.82%	KP773321.1
Escherichia coli isolate C929 quinolone resistance protein (qnrS2) gene, partial cds	1048	1048	8%	0.0	100.00%	KP773320.1
Photobacterium damselae strain Phdp Wu-1 plasmid plas1, complete sequence	987	987	12%	0.0	87.02%	CP018299.1
Aeromonas salmonicida strain Y47 plasmid pY47-3, complete sequence	950	1464	31%	0.0	78.11%	KT334398.1
Vibrio alginolyticus strain FDAARGOS_108 chromosome 1, complete sequence	907	1815	12%	0.0	86.10%	CP014053.1
Aeromonas sobria SNUFPC-A4 pAQ1-1 qnrS gene for quinolone resistance pentapeptide repeat protein QnrS5, complete CDS	881	881	9%	0.0	90.88%	NG_050546.1
Aeromonas sobria strain SNUFPC-A4 plasmid pAQ1-1 fluoroquinolone resistance protein gene, complete cds	881	881	9%	0.0	90.88%	HQ631377.1
Salmonella enterica subsp. enterica serovar Goldcoast strain Sal-5364 plasmid pSal-5364, complete sequence	870	870	12%	0.0	85.30%	CP039170.1
Salmonella enterica subsp. enterica serovar Goldcoast strain R18.0877 plasmid pR18.0877_278k, complete sequence	870	870	12%	0.0	85.30%	CP037959.1
Escherichia coli NIVEDI-P44 qnrS gene for quinolone resistance pentapeptide repeat protein QnrS13, complete CDS	870	870	10%	0.0	89.17%	NG_059275.1
Escherichia coli plasmid pM110_FII DNA, complete genome, isolate: M110	870	870	12%	0.0	85.30%	AP018140.1
Klebsiella pneumoniae isolate 833f714a-b38d-11e9-8998-68b599768938 genome assembly, plasmid: p13ARS_VSM0593-1	867	867	12%	0.0	85.18%	LR697125.1
Klebsiella pneumoniae isolate 8329a5f4-b38d-11e9-8998-68b599768938 genome assembly, plasmid: p13ARS_GMH0099	867	867	12%	0.0	85.18%	LR697099.1

Description	Max Score	Total Score	Query Cover	E value	Per. Ident	Accession
Salmonella enterica subsp. enterica serovar Newport strain 0945_2011 plasmid mediated quinolone resistance protein (qnrS2) gene, partial cds	867	867	6%	0.0	100.00%	KF649830.1

Graphic Summary

Alignments

Taxonomy

BLAST ® » **blastn suite** » results for RID-VGJD1CFV01R

Job Title	AY112998:Aeromonas veronii bv. Sobria class... ...
RID	VGJD1CFV01R Search expires on 10-30 22:16 pm
Program	BLASTN
Database	nt
Query ID	AY112998.1
Description	Aeromonas veronii bv. Sobria class B beta-lactamase (cephA3) gene, complete cds ...
Molecule type	nucleic acid
Query Length	1102

Descriptions

Description	Max Score	Total Score	Query Cover	E value	Per. Ident	Accession
Aeromonas veronii bv. Sobria class B beta-lactamase (cephA3) gene, complete cds	2036	2036	100%	0.0	100.00%	AY112998.1
Aeromonas veronii bv. sobria St46 cphA gene for subclass B2 metallo-beta-lactamase CphA3, complete CDS	1783	1783	87%	0.0	100.00%	NG_047666.1
Aeromonas veronii strain FC951 chromosome, complete genome	1751	1751	99%	0.0	95.63%	CP032839.1
Aeromonas veronii strain X11 chromosome, complete genome	1712	1712	99%	0.0	94.81%	CP024930.1
Aeromonas sp. CU5 chromosome, complete genome	1670	1670	92%	0.0	96.27%	CP023817.1
Aeromonas veronii strain X12 chromosome, complete genome	1663	1663	95%	0.0	95.32%	CP024933.1
Aeromonas veronii strain CB51, complete genome	1629	1629	97%	0.0	94.16%	CP015448.1
Aeromonas veronii strain TH0426, complete genome	1628	1628	92%	0.0	95.49%	CP012504.1
Aeromonas veronii strain 17ISAe chromosome, complete genome	1624	1624	97%	0.0	94.06%	CP028133.1
Aeromonas veronii strain MS-18-37 chromosome, complete genome	1594	1594	94%	0.0	94.33%	CP033604.1
Aeromonas hydrophila strain ZYAH72 chromosome, complete genome	1583	1583	91%	0.0	95.03%	CP016989.1
Aeromonas hydrophila strain GYK1, complete genome	1583	1583	91%	0.0	95.03%	CP016392.1
Aeromonas hydrophila strain D4, complete genome	1583	1583	91%	0.0	95.03%	CP013965.1
Aeromonas hydrophila strain JBN2301, complete genome	1583	1583	91%	0.0	95.03%	CP013178.1
Aeromonas hydrophila NJ-35, complete genome	1583	1583	91%	0.0	95.03%	CP006870.1
Aeromonas hydrophila J-1, complete genome	1583	1583	91%	0.0	95.03%	CP006883.1
Aeromonas hydrophila pc104A, complete genome	1583	1583	91%	0.0	95.03%	CP007576.1
Aeromonas hydrophila AL09-71, complete genome	1583	1583	91%	0.0	95.03%	CP007566.1
Aeromonas hydrophila ML09-119, complete genome	1583	1583	91%	0.0	95.03%	CP005966.1
Aeromonas veronii strain FDAARGOS_632 chromosome, complete genome	1565	1565	94%	0.0	93.84%	CP044060.1
Aeromonas veronii strain AVNIH1, complete genome	1554	1554	99%	0.0	92.36%	CP014774.1
Aeromonas veronii B565, complete genome	1539	1539	94%	0.0	93.37%	CP002607.1

Description	Max Score	Total Score	Query Cover	E value	Per. Ident	Accession
Aeromonas allosaccharophila ATCC 51208 class B beta-lactamase (cphA4) gene, complete cds	1474	1474	87%	0.0	94.21%	AY227050.1
Aeromonas allosaccharophila ATCC 51208 cphA gene for subclass B2 metallo-beta-lactamase CphA4, complete CDS	1452	1452	84%	0.0	94.66%	NG_050397.1
Aeromonas dhakensis gene for CphA-type class B metallo-beta-lactamase, complete cds, strain: MDC47T	1330	1330	81%	0.0	93.33%	AB765398.1
Aeromonas hydrophila strain AL06-06, complete genome	1328	1328	87%	0.0	91.79%	CP010947.1
Aeromonas hydrophila strain 23-C-23 chromosome, complete genome	1317	1317	87%	0.0	91.58%	CP038465.1
Aeromonas hydrophila strain WCX23 chromosome, complete genome	1317	1317	87%	0.0	91.58%	CP038463.1
Aeromonas hydrophila strain WCX23 chromosome, complete genome	1317	1317	87%	0.0	91.58%	CP028418.1
Aeromonas dhakensis strain KN-Mc-6U21, complete genome	1301	1301	81%	0.0	92.77%	CP023141.1
Aeromonas hydrophila strain MX16A chromosome, complete genome	1290	1290	87%	0.0	91.07%	CP018201.1
Aeromonas hydrophila strain AH10, complete genome	1290	1290	87%	0.0	91.06%	CP011100.1
Aeromonas hydrophyla cphA gene for metallo B-lactamase	1288	1288	82%	0.0	92.20%	X57102.1
Aeromonas sp. ASNIH4 chromosome, complete genome	1286	1286	81%	0.0	92.36%	CP026217.1
Aeromonas hydrophila subsp. dhakensis gene for CphA-type class B metallo-beta-lactamase, complete cds, strain: LMG19562	1258	1258	81%	0.0	91.89%	AB766134.1
Aeromonas hydrophila strain ZYAH75 chromosome, complete genome	1243	1243	84%	0.0	90.93%	CP016990.1
Aeromonas salmonicida strain A527 chromosome, complete genome	1243	1243	81%	0.0	91.56%	CP022550.1
Aeromonas hydrophila imiH gene for metallo beta-lactamase	1240	1240	81%	0.0	91.46%	AJ548797.1
Aeromonas hydrophila subsp. hydrophila strain WCHAH045096 chromosome, complete genome	1234	1234	87%	0.0	90.02%	CP028568.2
Aeromonas hydrophila AE036 cphA1 gene for subclass B2 metallo-beta-lactamase CphA1, complete CDS	1232	1232	78%	0.0	92.37%	NG_047667.1
Aeromonas hydrophila strain KN-Mc-1R2 chromosome, complete genome	1227	1227	82%	0.0	90.92%	CP027804.1
Aeromonas hydrophila GSH8-2 DNA, complete genome	1221	1221	81%	0.0	91.13%	AP019193.1
Aeromonas hydrophila strain AHNIH1, complete genome	1219	1219	78%	0.0	92.22%	CP016380.1
Aeromonas sp. CA23 chromosome, complete genome	1205	1205	81%	0.0	90.82%	CP023818.1
Aeromonas hydrophila subsp. hydrophila ATCC 7966, complete genome	1201	1201	81%	0.0	90.85%	CP000462.1
Aeromonas hydrophila cphA gene for ChpA family subclass B2 metallo-beta-lactamase ImiH, complete CDS	1186	1186	78%	0.0	91.44%	NG_050414.1
Aeromonas salmonicida subsp. salmonicida strain SHY16-3432 chromosome, complete genome	1181	1181	80%	0.0	90.57%	CP038102.1
Aeromonas salmonicida subsp. salmonicida 01-B526 chromosome, complete genome	1181	1181	80%	0.0	90.57%	CP027000.1
Aeromonas salmonicida subsp. salmonicida A449, complete genome	1181	1181	80%	0.0	90.57%	CP000644.1
Aeromonas sobria ATCC 43979 cphA gene for subclass B2 metallo-beta-lactamase CphA8, complete CDS	1179	1179	67%	0.0	95.06%	NG_050401.1

Description	Max Score	Total Score	Query Cover	E value	Per. Ident	Accession
Aeromonas veronii bv. veronii 224 cphA1 gene for subclass B2 metallo-beta-lactamase CphA1, complete CDS	1179	1179	67%	0.0	95.06%	NG_047670.1
Aeromonas veronii bv. veronii strain 224 class B beta-lactamase (cphA) gene, complete cds	1179	1179	67%	0.0	95.06%	AY261378.1
Aeromonas sobria strain ATCC 43979 class B beta-lactamase (cphA8) gene, complete cds	1179	1179	67%	0.0	95.06%	AY261375.1
Aeromonas veronii strain AQAV4 metallo-B-lactamase subclass B2 (cphA4) gene, partial cds	1164	1164	68%	0.0	94.67%	KM609958.1
Aeromonas veronii strain BCRC10375 class B beta-lactamase (cphA) gene, partial cds	1162	1162	64%	0.0	96.07%	JF972616.1
Aeromonas salmonicida strain O23A chromosome, complete genome	1155	1155	82%	0.0	89.67%	CP021654.1
Aeromonas jandaei ATCC 49568 cphA gene for subclass B2 metallo-beta-lactamase CphA7, complete CDS	1153	1153	69%	0.0	93.86%	NG_050400.1
Aeromonas jandaei ATCC 49568 class B beta-lactamase (cphA7) gene, complete cds	1153	1153	69%	0.0	93.86%	AY227053.1
Aeromonas salmonicida subsp. pectinolytica 34mel chromosome, complete genome	1147	1257	87%	0.0	90.64%	CP022426.1
Aeromonas hydrophila cphA gene for CphA-type class B metallo-beta-lactamase, complete cds, strain: RYU_N27	1136	1136	69%	0.0	93.46%	LC270625.1
Aeromonas veronii ATCC 49904 cphA1 gene for subclass B2 metallo-beta-lactamase CphA1, complete CDS	1134	1134	67%	0.0	94.01%	NG_047669.1
Aeromonas veronii strain ATCC 49904 class B beta-lactamase (cphA) gene, complete cds	1134	1134	67%	0.0	94.01%	AY261377.1
Aeromonas veronii bv. veronii ATCC 35624 cphA gene for subclass B2 metallo-beta-lactamase CphA6, complete CDS	1125	1125	69%	0.0	93.21%	NG_050399.1
Aeromonas veronii ATCC 35624 class B beta-lactamase (cphA6) gene, complete cds	1125	1125	69%	0.0	93.21%	AY227052.1
A.veronii imiS gene	1120	1120	78%	0.0	90.16%	Y10415.1
Aeromonas aquariorum strain A2-094 class B beta-lactamase (cphA) gene, partial cds	1105	1105	63%	0.0	95.02%	JF972624.1
Aeromonas veronii 163a cphA gene for ChpA family subclass B2 metallo-beta-lactamase ImiS, complete CDS	1099	1099	77%	0.0	90.04%	NG_050415.1
Aeromonas aquariorum strain A2-159 class B beta-lactamase (cphA) gene, partial cds	1088	1088	64%	0.0	94.13%	JF972628.1
Aeromonas hydrophila strain AQAH5 metallo-beta-lactamase subclass B2 (blaCphAH5) gene, complete cds	1075	1075	69%	0.0	92.03%	KP771880.1
Aeromonas aquariorum strain A2-013 class B beta-lactamase (cphA) gene, partial cds	1064	1064	63%	0.0	93.90%	JF972619.1
Aeromonas aquariorum strain BCRC17946 class B beta-lactamase (cphA) gene, partial cds	1057	1057	65%	0.0	93.07%	JF972618.1
Aeromonas aquariorum strain A2-070 class B beta-lactamase (cphA) gene, partial cds	1038	1038	63%	0.0	93.31%	JF972623.1
Aeromonas hydrophila AER 19 cphA gene for subclass B2 metallo-beta-lactamase CphA2, complete CDS	1037	1037	69%	0.0	91.11%	NG_050396.1
Aeromonas hydrophila metallo-beta-lactamase (cphA2) gene, complete cds	1037	1037	69%	0.0	91.11%	U60294.1
Aeromonas hydrophila subsp. hydrophila 212 cphA1 gene for subclass B2 metallo-beta-lactamase CphA1, complete CDS	1035	1035	67%	0.0	91.59%	NG_047671.1
Aeromonas hydrophila subsp. hydrophila strain 212 class B beta-lactamase (cphA) gene, complete cds	1035	1035	67%	0.0	91.59%	AY261379.1

Description	Max Score	Total Score	Query Cover	E value	Per. Ident	Accession
Aeromonas aquariorum strain A2-157 class B beta-lactamase (cphA) gene, partial cds	1033	1033	63%	0.0	93.17%	JF972627.1
Aeromonas hydrophila strain KN-MC-6U2 class B metallo-beta-lactamase (cphA) gene, partial cds	1024	1024	64%	0.0	92.57%	MK415753.1
Aeromonas aquariorum strain A2-107 class B beta-lactamase (cphA) gene, partial cds	1024	1024	65%	0.0	92.24%	JF972626.1
Aeromonas aquariorum strain A2-056 class B beta-lactamase (cphA) gene, partial cds	1022	1022	63%	0.0	92.89%	JF972621.1
Aeromonas aquariorum strain A2-042 class B beta-lactamase (cphA) gene, partial cds	1022	1022	63%	0.0	92.89%	JF972620.1
Aeromonas salmonicida ATCC 33658 cphA gene for subclass B2 metallo-beta-lactamase CphA5, complete CDS	1011	1011	68%	0.0	90.95%	NG_050398.1
Aeromonas salmonicida ATCC 33658 class B beta-lactamase (cphA5) gene, complete cds	1011	1011	68%	0.0	90.95%	AY227051.1
Aeromonas dhakensis strain KN-MC-6U21 class B metallo-beta-lactamase (cphA) gene, partial cds	1009	1009	65%	0.0	92.05%	MK415754.1
Aeromonas aquariorum strain A2-061 class B beta-lactamase (cphA) gene, partial cds	1007	1007	64%	0.0	92.15%	JF972622.1
Aeromonas aquariorum strain A2-098 class B beta-lactamase (cphA) gene, partial cds	1005	1005	63%	0.0	92.46%	JF972625.1
Aeromonas hydrophila subsp. hydrophila strain BCRC13018 class B beta-lactamase (cphA) gene, partial cds	1000	1000	63%	0.0	92.32%	JF972617.1
Aeromonas hydrophila strain KN-MC-5R2 class B metallo-beta-lactamase (cphA) gene, partial cds	994	994	63%	0.0	92.18%	MK415752.1
Aeromonas hydrophila strain KN-MC-5R1 class B metallo-beta-lactamase (cphA) gene, partial cds	994	994	63%	0.0	92.18%	MK415751.1
Aeromonas hydrophila strain KN-MC-4N3 class B metallo-beta-lactamase (cphA) gene, partial cds	989	989	63%	0.0	92.03%	MK415750.1
Aeromonas hydrophila strain KN-MC-4N1 class B metallo-beta-lactamase (cphA) gene, partial cds	989	989	63%	0.0	92.03%	MK415749.1
Aeromonas hydrophila strain KN-MC-2R1 class B metallo-beta-lactamase (cphA) gene, partial cds	989	989	63%	0.0	92.03%	MK415748.1
Aeromonas hydrophila strain KN-MC-10N1 class B metallo-beta-lactamase (cphA) gene, partial cds	977	977	64%	0.0	91.33%	MK415756.1
Aeromonas hydrophila strain KN-MC-1R1 class B metallo-beta-lactamase (cphA) gene, partial cds	974	974	61%	0.0	92.50%	MK415746.1
Aeromonas hydrophila strain KN-MC-1R2 class B metallo-beta-lactamase (cphA) gene, partial cds	968	968	61%	0.0	92.35%	MK415747.1
Aeromonas dhakensis DNA, similar to CphA-type class B metallo-beta-lactamase, strain: A2-155	965	1335	81%	0.0	94.42%	AB765405.1
Aeromonas salmonicida subsp. masoucida strain RFAS1 chromosome, complete genome	953	1198	80%	0.0	90.96%	CP017143.1
Aeromonas salmonicida strain S68, complete genome	953	1198	80%	0.0	90.96%	CP022186.1
Aeromonas salmonicida strain S44, complete genome	953	1198	80%	0.0	90.96%	CP022181.1
Aeromonas salmonicida strain S121, complete genome	953	1198	80%	0.0	90.96%	CP022175.1

Graphic Summary
